# Rad59-Facilitated Acquisition of Y′ Elements by Short Telomeres Delays the Onset of Senescence

**DOI:** 10.1371/journal.pgen.1004736

**Published:** 2014-11-06

**Authors:** Dmitri Churikov, Ferose Charifi, Marie-Noëlle Simon, Vincent Géli

**Affiliations:** Marseille Cancer Research Center (CRCM), U1068 Inserm, UMR7258 CNRS, Aix Marseille University, Institut Paoli-Calmettes, LNCC (Equipe labellisée), Marseille, France; University of Georgia, United States of America

## Abstract

Telomerase-negative yeasts survive via one of the two Rad52-dependent recombination pathways, which have distinct genetic requirements. Although the telomere pattern of type I and type II survivors is well characterized, the mechanistic details of short telomere rearrangement into highly evolved pattern observed in survivors are still missing. Here, we analyze immediate events taking place at the abruptly shortened VII-L and native telomeres. We show that short telomeres engage in pairing with internal Rap1-bound TG_1–3_-like tracts present between subtelomeric X and Y′ elements, which is followed by BIR-mediated non-reciprocal translocation of Y′ element and terminal TG_1–3_ repeats from the donor end onto the shortened telomere. We found that choice of the Y′ donor was not random, since both engineered telomere VII-L and native VI-R acquired Y′ elements from partially overlapping sets of specific chromosome ends. Although short telomere repair was associated with transient delay in cell divisions, Y′ translocation on native telomeres did not require Mec1-dependent checkpoint. Furthermore, the homeologous pairing between the terminal TG_1–3_ repeats at VII-L and internal repeats on other chromosome ends was largely independent of Rad51, but instead it was facilitated by Rad59 that stimulates Rad52 strand annealing activity. Therefore, Y′ translocation events taking place during presenescence are genetically separable from Rad51-dependent Y′ amplification process that occurs later during type I survivor formation. We show that Rad59-facilitated Y′ translocations on X-only telomeres delay the onset of senescence while preparing ground for type I survivor formation.

## Author Summary

In humans, telomerase is expressed in the germline and stem, but is repressed in somatic cells, which limits replicative lifespan of the latter. To unleash cell proliferation, telomerase is reactivated in most human cancers, but some cancer cells employ alternative lengthening of telomeres (ALT) based on homologous recombination (HR) to escape senescence. Recombination-based telomere maintenance similar to ALT was originally discovered in budding yeast deficient in telomerase activity. Two types of telomere arrangement that depend on two genetically distinct HR pathways (RAD51- and RAD59-dependent) were identified in post-senescent survivors, but the transition to telomere maintenance by HR is poorly understood. Here, we show that one of the earliest steps of short telomere rearrangement in telomerase-negative yeast is directly related to the “short telomere rescue pathway” proposed 20 years ago by Lundblad and Blackburn, which culminates in the acquisition of subtelomeric Y′ element by shortened telomere. We found that this telomere rearrangement depends on Rad52 strand annealing activity stimulated by Rad59, thus it is distinct from Rad51-dependent Y′ amplification process observed in type I survivors. We show that continuous repair of critically short telomeres in telomerase-negative cells delays the onset of senescence and prepares the ground for telomere maintenance by HR.

## Introduction

Telomeres are nucleoprotein structures found at the physical ends of chromosomes. Their terminal location defines their two main functions: protection of the chromosome ends from illegitimate repair reactions and prevention of the loss of terminal DNA due to either degradation or incomplete replication [1]. In *Saccharomyces cerevisiae*, the first function is accomplished primarily by Rap1, which wraps tandem telomeric DNA repeats to inhibit NHEJ [2], and recruits Rif1 and Rif2 to restrain MRX-mediated 3′ end resection [3], [4], thus limiting recruitment of HR factors and checkpoint signaling [5]. The second function is mediated by Cdc13 bound to the single-stranded G-rich 3′ overhang at the extreme terminus of a telomere. Cdc13 forms alternative complexes with either Est1 or Stn1-Ten1 to coordinate telomerase-mediated synthesis of the G-rich strand with the synthesis of the complementary strand by DNA polymerase α [6].

As in mammals, telomeres in yeast cells with reduced telomerase activity progressively shorten with each cell division until they are recognized as DNA damage and recruit Mec1 kinase that initiates irreversible G2/M arrest [7]–[9]. At the level of cell population, telomere dysfunction is manifested as crisis, when majority of the cells irreversibly arrest in G2/M [7]. Most of the cells die, but at low frequency survivors emerge, which maintain their telomeres via recombination [10], [11], implying that homologous recombination (HR) can serve as a bypass pathway to sustain viability in the absence of telomerase.

The survivors are classified in two types based on their telomere arrangement and growth characteristics [12], [13]. The type I survivors have tandem arrays of subtelomeric Y′ elements separated by short tracts of TG_1–3_ repeats at most chromosome ends, and also short terminal TG_1–3_ repeats [10]. Their growth is interrupted by frequent periods of arrest and in the competitive conditions of liquid culture they are outcompeted by the more robust type II survivors. In type II survivors, terminal TG_1–3_ repeats are abnormally elongated and are very heterogeneous in length. It is believed that they are established by stochastic lengthening events that likely involve rolling circle replication [14]. *RAD52* is required for generation of both types of survivors. *RAD51*, *RAD54*, *RAD57* are specifically required to generate type I, whereas type II survivors depend on MRX complex, *RAD59* and *SGS1*, encoding the only RecQ helicase in yeast [13], [15], [16]. In addition, *POL32* encoding a non-essential subunit of DNA polymerase δ is required for generation of both survivor types, implying involvement of the processive repair DNA synthesis in the recombination-based telomere rearrangements [17], [18]. Recently, a genome-wide screen aimed to identify telomere-length-maintenance genes that regulate telomere structure in post-senescence survivors unveil new regulators of Type I and II recombination [19]. Notably, Type I recombination was shown to depend on the helicase Pif1 and on the chromatin remodelling complex INO80.

Although genetic requirements for the formation of two types of survivors and their telomere patterns have been well characterized, much less is known about actual recombination events that lead to reorganization of the original short telomere into the patterns observed in survivors. In budding yeast, telomere shortening does not cause end-to-end chromosome fusions, as does the removal of Rap1 from telomeres [2]; instead, gene conversion increases near short telomeres indicating de-repressed recombination [20]. There is a controversy, however, whether type II recombination preferentially takes place at long or short telomeres [14], [21], [22]. Little is known about the telomere length preference of type I pathway. Early studies looking at the propagation of linear plasmids in yeast uncovered that they can recombine with the yeast chromosome ends and acquire telomere-adjacent sequences called Y′ elements [23]. Y′ elements found at many chromosome ends fall into two size classes, 6.7 (Y′-L) and 5.2 (Y′-S) kb-long, that differ by a 1.5 kb insertion/deletion [24]. Another subtelomeric sequences called X elements are present at all chromosome ends immediately proximal to either Y's or terminal TG_1-3_ repeats when Y′ is absent. The junction between X and Y′ elements often, but not always, contain short tracts of TG_1–3_ repeats [24], [25]. Importantly, only the Y′ and not X elements can be transferred on linear plasmids, and this is mediated by recombination between the terminal TG_1–3_ repeats added onto the plasmid ends by telomerase and the internal TG_1–3_ tracts present between the X and Y′ elements [23]. Pioneering work of Lundblad and Blackburn showed that *est1*Δ survivors arose as the result of the acquisition of Y′ elements by X-only telomeres and amplification of these elements on many chromosome ends. They proposed a model of telomere rescue via a recombination event between the terminal TG_1–3_ repeats of one telomere and an internal TG_1–3_ tract in another [10].

We have previously demonstrated using single cell analysis that Rad52-containing foci are assembled at the telomeres in a length-dependent manner in presenescent cells many generations before the onset of senescence [26]. The recruitment of recombination factors to short telomeres is in accord with increased recombinogenic activity of short telomeres observed in both yeast [20] and mammals [27]. Of note, inactivation of HR, particularly via deletion of *RAD52* and *RAD51* (but to a much lesser extent of *RAD59*), causes early decline in proliferative capacity of telomerase-negative yeast indicating that telomere maintenance most likely becomes dependent on HR soon after telomerase inactivation [28], [29]. Surprisingly, the rate of telomere shortening (population average length) is unaffected in HR-deficient yeast. All these observations raise the question of telomere recombination dynamics in presenescent cells, the mechanism of Y′ acquisition by X-only telomeres and the role of recombination proteins in maintaining telomerase-negative strains alive during presenescence. Another unresolved issue is whether a single critically short telomere is sufficient to induce cell cycle arrest. Complete loss of a single telomere causes Rad9-dependent arrest even in telomerase-proficient cells [30]. This does not seem to be the case when a very short telomere is created in telomerase negative cells [8], [28], but the fate of this abruptly shortened telomere remains obscure. In this study, we aimed to characterize the primary recombination event that takes place at short telomeres in the absence of telomerase. To this end we put together a system to simultaneously shorten modified VII-L telomere and inactivate telomerase. Bulk liquid cultures turned out to be inappropriate to address the fate of the abruptly shortened VII-L telomere, so we adapted clonal analysis. We found that the subtelomereless VII-L end acquired Y′ element in clonal populations originated from transiently arrested cells. Cloning and sequencing of the Y′ translocation junctions from multiple clones revealed that Y′ acquisition was initiated by recombination between the short terminal TG_1–3_ repeats at VII-L and the Rap1-bound internal TG_1–3_-like tracts present between X and Y′ elements on other chromosome ends. Such recombination initiates Pol32-dependent BIR, which results in non-reciprocal translocation of the entire Y′ element and terminal TG_1–3_ tract from the chromosome-donor onto the shortened telomere. Surprisingly, Y′ translocation events were Rad51-independent, but were instead promoted by Rad59 that stimulates Rad52 strand annealing activity. We found that the same mechanism operates at short native X-only telomeres, but it is much more efficient since translocated Y's are readily detectable in bulk liquid cultures during presenescence. In addition, sequence composition of the translocation junctions is simpler at native telomere VI-R, indicating that Y′ translocation on a native end is relatively straightforward event. We further show that *RAD59* deletion compromises the efficiency of Y′ translocation on native telomere XV-L, and results in both accelerated senescence and prolonged crisis. Our results extend the model of short telomere rescue proposed by Lundblad and Blackburn more than 20 years ago [10], and they reinforce the notion that it is the overall depletion of the TG_1–3_ repeats on multiple chromosome ends rather than abrupt shortening of a few telomeres that defines the onset of senescence.

## Results

### VII-L telomere shortened beyond 60 bp becomes unstable and causes transient arrest

**Figure 1:**
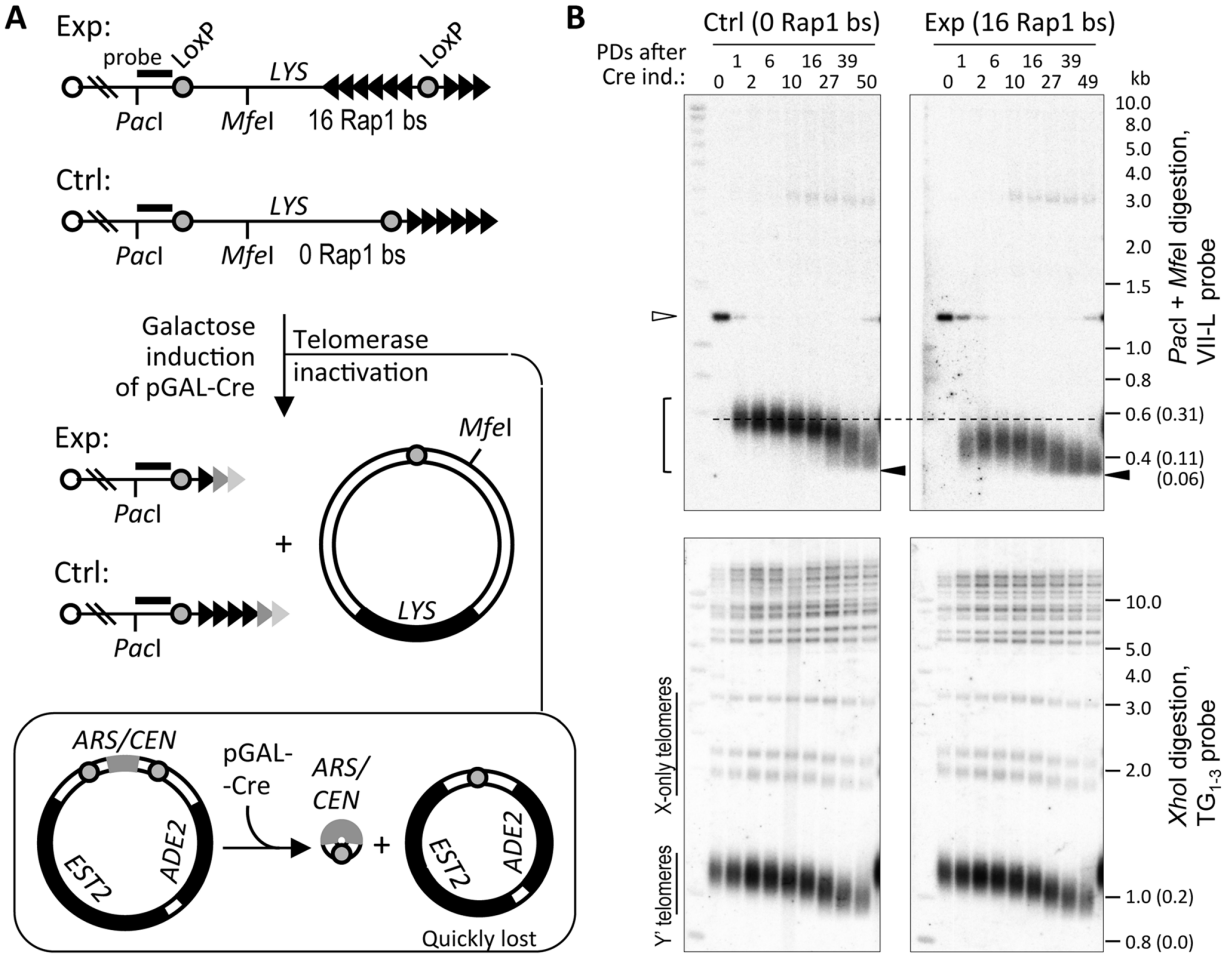
Telomere shortening reaches the limit at ∼60 bp of TG_1–3_ repeats. (A) Schematic of the Cre-loxP recombination-based system for abrupt shortening of the TelVII-L. Addition of telomere repeats by telomerase was blocked by simultaneous deletion of the only *EST2* gene copy along with *ADE2* marker by Cre-loxP recombination from the ARS/CEN plasmid (double Cre-loxP system). Fading triangles represent gradual loss of telomere repeats. Exp and Ctrl denote TelVII-L in the experimental and control strains, respectively, which differ by the presence/absence of the 16 Rap1-binding sites (bs) flanked by the loxP sites. (B) Southern blot analysis of VII-L (top panels) and bulk (bottom panels) telomeres in the control and experimental strains that differ in the length of the single telomere VII-L. The cells grown in S-raffinose -Lys were shifted to galactose to induce Cre expression, which simultaneously induced recombination at VII-L and *EST2* deletion from the plasmid. DNA extracted from the samples taken at indicated PDs after Cre induction was digested with either *Pac*I or *Mfe*I (top panels) or *Xho*I (bottom panels) and subjected to Southern blot analysis with either VII-L-specific (shown in A) or TG_1–3_ probe to visualize VII-L and bulk telomeres, respectively. Open arrowhead indicates *Pac*I-*Mfe*I fragment and the bracket indicates terminal *Pac*I fragments of the TelVII-L before and after Cre-loxP recombination, respectively. Note that a fuzzy band that appears at 10 PD and migrates just below 3 kb (top panels) is most likely resected VII-L terminal fragment which is largely single-stranded. Closed arrowhead points out the limit of telomere shortening. Numbers given in parentheses next to molecular weights are the lengths of the TG_1–3_ repeat tracts obtained by subtracting the non-telomeric portion of the terminal fragments. *Xho*I cuts once within each Y′ element, ∼850 bp away from terminal TG_1–3_ repeats, thus releasing a terminal fragment containing telomere. These so called Y′ telomeres migrate at the bottom of the gel, whereas X-only telomeres migrate above them.

To address the processing of a short telomere without Y′ elements in the absence of telomerase, we employed the site-specific recombination system to induce abrupt shortening of a single telomere [31]. In this system, Cre induction causes excision of the basal portion of the telomere VII-L (TelVII-L) flanked by loxP sites (Figure 1A). In the presence of telomerase, shortened telomere is extended until its length returns to equilibrium [32]. To examine how this telomere will be processed in the absence of telomerase, we combined the abrupt shortening of TelVII-L with an inducible deletion of the plasmid-borne *EST2* [33].

As expected, inactivation of telomerase completely abolished elongation of the TelVII-L after it was shortened via Cre-loxP recombination. Instead, its length decreased further until the bottom of the telomere length distribution reached a defined limit beyond which no shortening was observed (Figure 1B). The lower tail of the TelVII-L length distribution in the control strain also reached the same limit albeit with a delay of ∼20 population doublings (PDs) consistent with its greater initial length. We estimated that the lower limit of the TelVII-L length distribution corresponds to ∼60 bp of TG_1–3_ repeats (Figure S1). Since the probe anneals to the unique sequence of the terminal *Pac*I fragment (Figure 1A), even complete loss of TG_1–3_ repeats should not affect hybridization signal. Thus, we reasoned that shortening of the TG_1–3_ tract beyond 60 bp causes the elimination of cells with critically short telomeres from the exponentially growing culture propagated via serial dilutions.

### Clonal analysis of the telomerase-negative cells reveals VII-L end rearrangement consistent with Y′ element translocation

**Figure 2:**
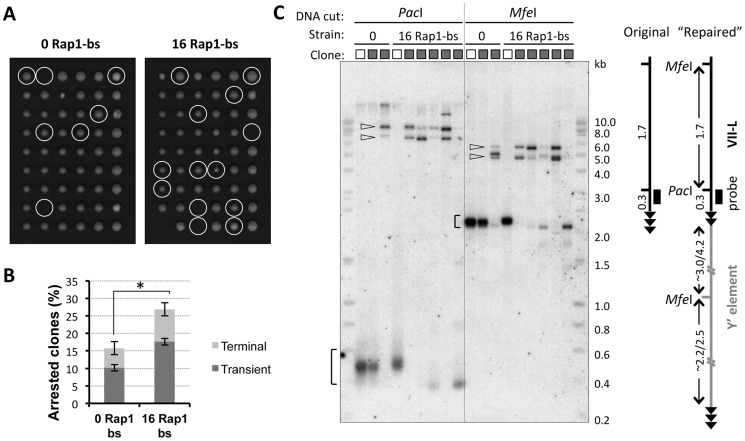
Clones derived from transiently arrested cells exhibit VII-L end rearrangement consistent with Y′ element translocation. (A) Microcolony formation assay was performed to identify cells undergoing transient arrest after telomerase inactivation. Single cells from two double Cre-loxP strains that differ in a length of TelVII-L were micromanipulated onto a grid on YPD agar plate at 36 h (∼15 PD) after induction of Cre expression in liquid culture. Cell divisions were monitored microscopically and the numbers of cells in microcolonies were counted at 4 and 6 h after plating. Representative images of the plates taken after 3 days of colonies outgrowth are shown. The positions where cell division arrest was detected at the time of plating are circled. (B) Histogram showing the fraction of cells which arrested divisions either during the first or second cell cycle after plating. Mean values ±SE for two independent micromanipulations at 15 and 18 PD after Cre induction are shown. One-sided chi-square test was used to evaluate the significance of the difference in overall fraction of arrested cells. χ^2^ (1, N = 162)  = 3.08, p = 0.040. (C) Southern blot analysis of the VII-L end in the clones derived from cells recovered from transient arrest (A). The lanes marked with white and grey boxes correspond to “no delay” control and “growth-delayed” clones, respectively. The colonies were inoculated in 3 ml of YPD and cultured overnight to generate sufficient number of cells for DNA extraction. For each clone two aliquots of DNA were digested separately with *Pac*I and *Mfe*I, which recognition site positions at the VII-L end are shown in the diagram. Digested DNA was subjected to Southern blot analyses with VII-L-specific probe (black rectangle in the scheme). The brackets indicate terminal fragments of the TelVII-L, whereas open arrowheads point to the high molecular weight fragments resulted from VII-L end rearrangement.

To isolate the cells undergoing cell cycle arrest due to TG_1–3_ tract shortening beyond the 60 bp threshold, we conducted clonal analysis of the telomerase-negative cultures at ∼15 PD after Cre induction. To this end, single cells were micromanipulated on a grid of agar, and analyzed for their ability to form microcolonies. While many cells divided regularly, at least once every 2 hours, and formed microcolonies of more than 16 cells after 8 hours on agar, a fraction of cells never divided during this time or stopped dividing at the 2- or 4-cell stage. These arrested microcolonies were marked (Figure 2A). Unexpectedly, most of the cells, which initially failed to divide, formed colonies after four days at 30°C (Figure 2A). Therefore, the majority of cells was able to overcome cell cycle arrest and resumed divisions. The fraction of arrested cells was significantly greater in the strain with shortened TelVII-L compared to the control strain (Figure 2B and Figure S2) indicating that shortening of a single telomere aggravated the effect of telomerase inactivation on cell cycle progression.

The state of the TelVII-L in clonal populations was analyzed by Southern blotting (Figure 2C). The terminal VII-L fragments with typical smeary appearance were detected in the expected size range for the control (no arrest) clones. In contrast, most of the clones that had undergone transient arrest completely lost the VII-L signal in this range. Instead, much larger fragments hybridized with VII-L-specific probe (Figure 2C), suggesting that VII-L end has been rearranged. These larger fragments grouped in two size classes after digestion with either *Pac*I or *Mfe*I, and were remarkably consistent with Y′ element translocation (see schematic in Figure 2C). Grouping of the fragments in two size classes could be well explained by translocation of either long (Y′-L) or short (Y′-S) version of the Y′ elements, which differ by a 1.5 kb insertion/deletion [24]. Most of the clones showed the presence of both Y′ classes. This heterogeneity is likely created due to independent acquisition of Y′ elements by TelVII-L in different cells (2- or 4-cell stage arrest), or even by two sister TelVII-L chromatids within a G2-arrested cell. Thus, we were able to isolate subclones descended from single recombination events (group A in Figure S2) by sequential micromanipulation of the cells as they came out of the arrest. Those transiently arrested clones that did not show VII-L end rearrangement (Figure 2C and Figure S2) could have either repaired it by addition of the terminal TG_1–3_ repeats [22] or arrested due to shortening of one of the native telomeres.

### Y′ element is preferentially translocated on short telomeres

**Figure 3:**
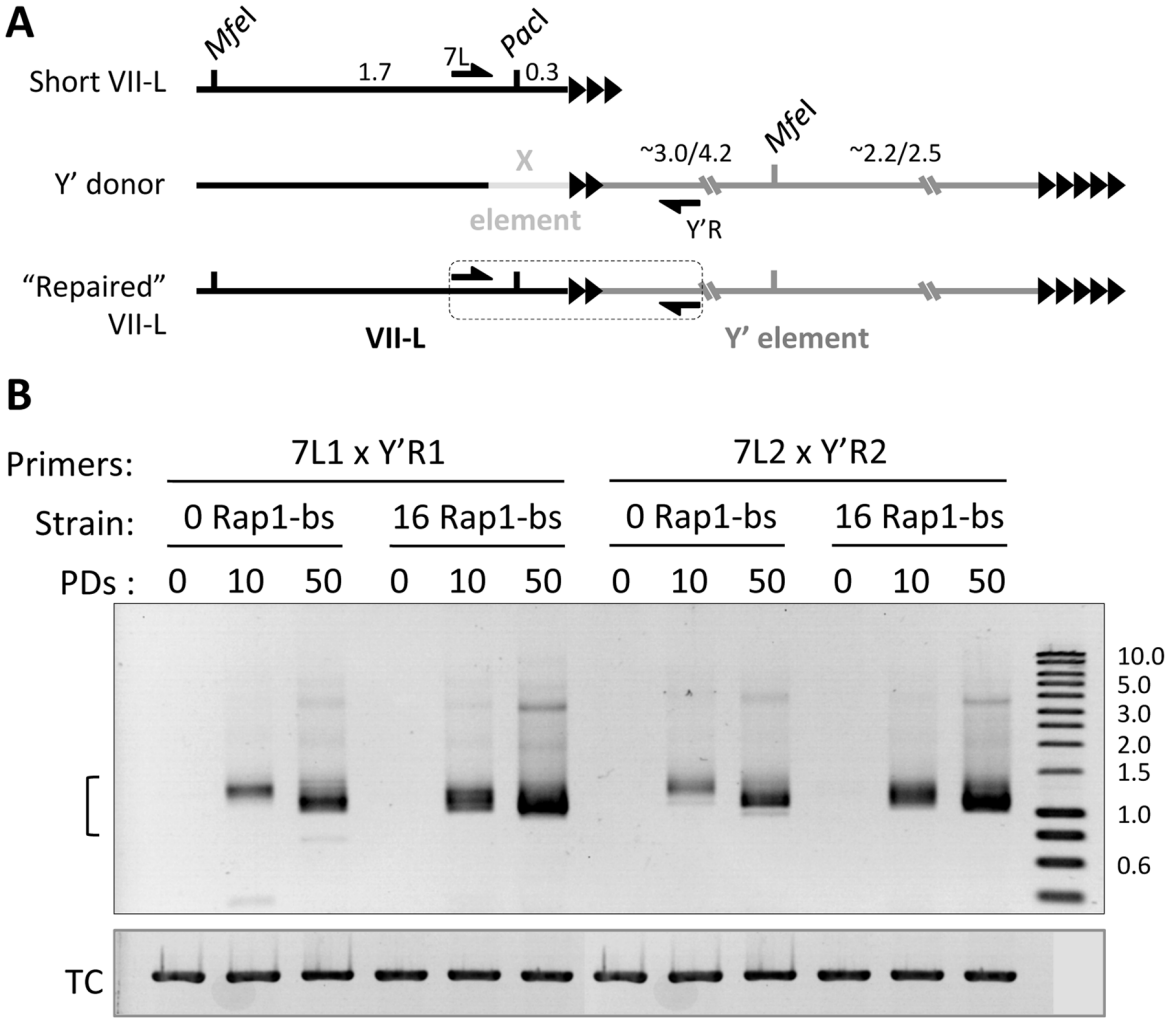
Y′ element translocation on VII-L end is readily detected by PCR across the junction. (A) Schematic of the primer design for detection of the Y′ translocation onto modified VII-L end. Y′ donor refers to any X and Y′ element-containing native chromosome end. (B) Semi-quantitative PCR across the VII-L/Y′ junction. DNA was extracted from double Cre-loxP strains grown in liquid culture at 0, 10, 50 PD after induction of Cre expression (see the kinetics of telomere shortening in Figure 1B) and used as a template in PCR reaction with two different pairs of primers designed to amplify the VII-L/Y′ junction shown in (A). The bracket indicates expected size range of the junction PCR product. TC – template control, PCR at the *NUP60* locus. See also Figure S8 for Y′ translocation on the native telomere VI-R.

To verify the hypothesis of Y′ translocation and the kinetics of this repair process, we designed primer pairs to amplify the putative junction region between the VII-L end and the Y′ element (Figure 3A, Table S1). We analyzed the presence of the junction PCR product at 0, 10, and 50 PDs after inactivation of telomerase in the strain bearing a critically short telomere (16 Rap1-bs) and in the control strain (0 Rap1-bs) (Refer to Figure 1B). We detected the junction-specific PCR product at VII-L (Figure 1A) as early as 10 PD in liquid culture after inactivation of telomerase and telomere shortening. The intensity and heterogeneity of the junction PCR product increased dramatically by 50 PD (Figure 3B). Moreover, the junction PCR product was more intense at 10 PD in the strain with short telomere compared to that in the control strain. When the same PCR approach to detect Y′ translocation was performed on the native VI-R end, the appearance of recombined telomere followed the same kinetics in the “0” and “16 Rap1-bs” strains (see further).

To assess the relationship between telomere length and Y′ acquisition in a more quantitative fashion, we analyzed the frequency of Y′ translocation on TelVII-L by Southern blotting in the random clones isolated from “0” and “16 Rap1-bs” strains. This analysis revealed significantly greater fraction of clones with Y′ element translocated on TelVII-L in the “16 Rap1-bs” (10/18) as compared to that in “0 Rap1-bs” (1/19) strain when the clones were isolated at 18 PD after Cre induction (Fisher's exact test P value 0.001; Figure S2). The same tendency was observed when the clones were isolated at 12 PD after Cre induction, but the frequency of Y′ translocation at this earlier time was too low for statistical evaluation (0/24 in “0 Rap1-bs” and 3/24 in “16 Rap1-bs” strain). In addition, the fraction of “16 Rap1-bs” clones with Y′ translocated on TelVII-L was significantly greater when the clones were isolated at 18 as compared to 12 PD after Cre induction (Fisher's exact test P value 0.006; Figure S2). The frequency of Y′ translocation among “0 Rap1-bs” clones was still low at these time points for statistical evaluation. We concluded that Y′ element is preferentially translocated on short telomeres; and the fraction of cells with translocated Y′ element grows with time after telomerase inactivation.

### Y′ translocation is mediated by homeologous recombination between the terminal and internal TG_1–3_ repeats

Cloning and sequencing of the junction PCR products revealed that they are in fact composed of the joint VII-L and Y′ element sequences, thereby confirming Y′ translocation on the VII-L end. Remarkably, all clones contained the TG_1–3_ repeats at the breakpoint between the VII-L and Y′ sequences, consistent with our assumption that Y′ translocation was initiated by VII-L terminal repeats, which recombined with internal TG_1–3_ tracts located between the subtelomeric X and Y′ elements of the chromosome donor. Sequence analysis of the Y′ segments of the junction clones revealed that the choice of the donor for Y′ translocation was not random: the chromosome arms VI-L and VII-R were used most frequently as the donors (Table 1).

**Table 1:** Summary of the distribution of Y′ elements among chromosome ends, subtelomeric Rap1 binding (ChIP-on-chip), and Y′ translocation frequencies.

Chromosome arm	Y' element type	Rap1 binding peak (kb)*	Subtelomeric TG_1–3_ **	Translocation counts on	
				VII-L	VI-R
I-L	-	-			
I-R	-	-			
II-L	Short	5.8	degener.		
II-R	-	-			
III-L	-	-			
III-R	-	-			
IV-L	-	-			
IV-R	Long	uncertain	97 bp		
V-L	Long	6.5	25 bp		
V-R	Long	6.9	degener.		
VI-L	Short	4.9	139 bp	18	4
VI-R	-	-			
VII-L	-	-			
VII-R	Long	6.8	degener.	13	4
VIII-L	Short	5.2	degener.		2
VIII-R	Short	6.0	160 bp	1	1
IX-L	Long	7.0	degener.		
IX-R	-	5.2			
X-L	Long	7.0	degener.		
X-R	-	-			
XI-L	-	-			
XI-R	-	-			
XII-L	Short tandem	5.8 and 11.6	64 and 163 bp		
XII-R	Long tandem	6.5 and 13.0	128 bp and degener.	1	
XIII-L	Short	5.7	52 bp		
XIII-R	-	-			
XIV-L	Long	6.7	75 bp		
XIV-R	-	-			
XV-L	-	-			
XV-R	Long	6.7	degener.		
XVI-L	Long	6.5	degener.		
XVI-R	Short	5.3	degener.		3

*The distance from terminal TG_1–3_ repeats.

**Sequence is considered degenerate if there is no TG_1–3_ stretches longer than 12 bp.

When perfect sequence is >12 bp, there could be still additional degenerate sequence between X and Y′ or between two Y′ elements.

**Figure 4:**
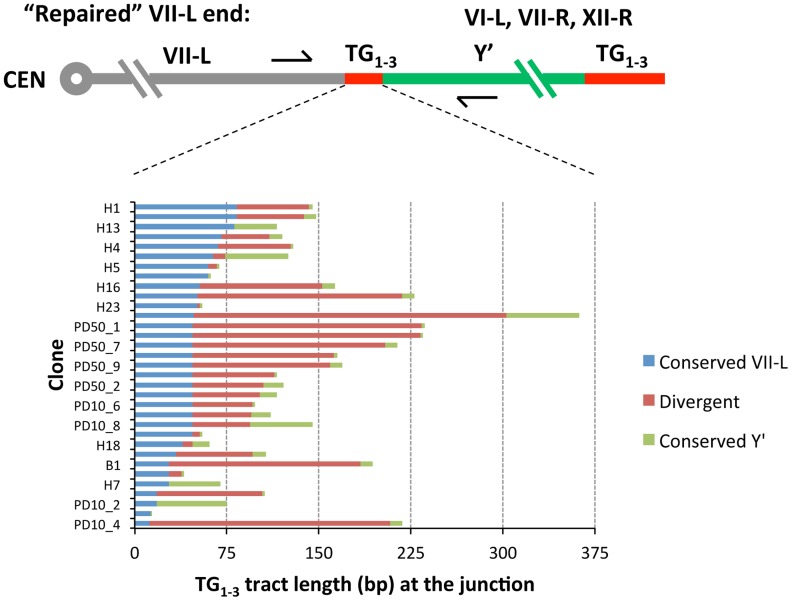
Sequence divergence within internal TG_1–3_ repeat tracts of the clones resulted from Y′ element translocation on the shortened VII-L end. DNA fragments encompassing VII-L/Y′ junctions were PCR-amplified from DNA of the “16 Rap1-bs” strain at either 10 or 50 PD after Cre induction, cloned and sequenced as described in Materials and Methods. The DNA sequences of the cloned VII-L/Y′ junctions were aligned at the VII-L side and the point of divergence was determined from sequence alignment. The sequences past the divergence point were analyzed by BLAST to identify the chromosomal origin of the Y′ element. Then the cloned junctions were aligned with the corresponding genomic sequences and the point of divergence from the Y′ element side was determined. The sequences identical at the VII-L side are in blue, and those identical at the donor telomeres are in green. Divergent sequences are in red. The donor telomeres are indicated.

To characterize recombination breakpoint we searched for the point of sequence divergence within TG_1–3_ repeats at the VII-L and Y′ junction. The exact pattern of irregular repeats newly synthesized by yeast telomerase differs between the molecules [34]. When telomere repeat sequences from clonal populations are aligned, only very distal 40–100 nt-long portions maintained by telomerase are divergent, whereas proximal regions are identical in sequence [35]. The divergent distal portion is quickly lost after telomerase inactivation. We found that TG_1–3_ repeats adjacent to the VII-L end shared 47±18 (SD) nt of identity before they diverged (Figures 4 and S3A), indicating that VII-L telomeres were at least that long at the time they engaged in recombination. Only in a few clones, the TG_1–3_ sequence past the divergence point continued without interruption by repeats found proximal to the acquired Y′ element (Figure S3B). In most of the clones, however, the repeats of the VII-L and Y′ origins were separated by divergent TG_1–3_ repeats (Figure 4). These intervening TG_1–3_ tracts varied in length and sometimes were quite long (e.g. 255 bp in the clone H10). We speculate that they might have resulted from abortive DNA repair synthesis due to rejection of homeologous heteroduplexes formed by TG_1–3_ repeats followed by template switch events [36]. Otherwise, the divergent regions at the junctions could be generated via recombination between two terminal TG_1–3_ repeat tracts, or even by residual telomerase activity during the first few PDs after Cre induction.

### *RAD51*-independent BIR accounts for the majority of Y′ translocations on VII-L end

**Figure 5:**
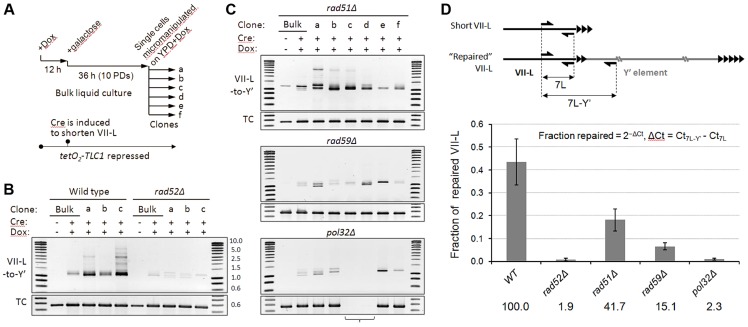
Y′ translocation depends on Rad52 and Pol32, is facilitated by Rad59, but is largely independent of Rad51. (A) Schematic of the experiment. (B and C) The semi-quantitative junction PCR assays for Y′ element translocation on VII-L end. Tet-off *TLC1* strains with indicated gene deletions were grown in the presence of Dox to suppress *TLC1* expression. Abrupt shortening of the TelVII-L was induced via transient induction of Cre by shifting cells to galactose for 36 h. At the end of Cre induction, single cells were micromanipulated onto a grid of YPD agar supplemented with Dox, and the formation of microcolonies was monitored microscopically. After four days of colonies outgrowth (Figure S5), DNA was extracted from clonal populations recovered from transiently arrested cells and from bulk populations grown in liquid culture for 48 h after Cre induction. PCR was performed with the primers designed to amplify the VII-L/Y′ junction (Figure 3A). TC, template control (PCR at the very terminus of VII-L). The bracket indicates two *pol32Δ* clones that lost the most terminal VII-L primer site due to VII-L end rearrangement that is distinct from Y′ translocation (see Figure S6). (D) Real-time quantitative PCR analysis of the genetic requirements of Y′ translocation. The fractions of “repaired” VII-L for each genotype were calculated as follows: fraction repaired  = 2^−ΔCt^, ΔCt = Ct_7L-Y_′−Ct_7L_, where 7L and 7L-Y′ represent amplification of the total and “repaired” VII-L as indicated in the schematic. Data are presented as mean ±SE for 15, 6, 18, 18, and 6 random clones of WT, *rad52Δ*, *rad51Δ*, *rad59Δ*, and *pol32Δ*, respectively, isolated at ∼16 PD after Cre induction. The numbers below the graph indicate the efficiencies of VII-L repair by Y′ translocation for each genotype relative to WT (set to 100%).

To get insight into the mechanism of short telomere repair, we examined its genetic requirements. To this end, we generated a set of isogenic mutants by deleting *RAD52*, *RAD51*, and *RAD59* genes in a strain with the VII-L telomere modified for inducible shortening and *TLC1* allele with a tetracycline-regulatable promoter (*tetO_2_-TLC1*). Upon addition of doxycycline (Dox), expression of *TLC1* is tightly repressed and telomeres shorten progressively with each generation ([26] and Figure S4). We first examined the effect of gene deletions on the efficiency of Y′ translocation by semi-quantitative PCR assay. Telomerase was inactivated by addition of Dox and abrupt shortening of the VII-L was then induced following the scheme in Figure 5A. We analyzed junction PCR products in clonal populations of HR-proficient or mutant cells (Figure 5B,C and Figure S5). As expected, robust junction PCR products indicating Y′ translocation events were detected in the clonal populations of HR-proficient cells (Figure 5B). Weaker product was also detectable in the mixed population of cells grown in liquid culture (Bulk). *RAD52* deletion nearly completely abolished Y′ translocation as judged by severe reduction of the amplified VII-L/Y′ junctions in either mixed or clonal populations (Figure 5B). Thus, Rad52 is essential for recombination between TG_1–3_ repeats that leads to Y′ translocation. Surprisingly, deletion of the *RAD51* had little effect on the efficiency of Y′ translocation (Figure 5C, top panel), demonstrating that Rad51 may not be essential for recombination between TG_1–3_ repeats. In contrast, *RAD59* deletion reduced both the amount and the heterogeneity of amplified VII-L/Y′ junctions (Figure 5C, midpanel) suggesting lower frequency of TG_1–3_ repeat recombination in the absence of Rad59. These results indicate that Rad51 filament assembly may not be required at the 3′ overhang of the short telomere, which pairing with an internal tract of the TG_1–3_ repeats could depend only on Rad52 strand annealing activity which is stimulated by Rad59.

While Y′ translocation is initiated by TG_1–3_ repeat recombination, its completion likely depends on break induced replication (BIR), a pathway used to repair a DSB when homology is limited to its one side [37]. Deletion of *POL32*, encoding a nonessential subunit of Polδ required for processive DNA synthesis during BIR [38], severely reduced the efficiency of Y′ translocation as evidenced by drastically reduced junction PCR product (Figure 5C, bottom panel). Notably, the PCR at the VII-L terminus, which served as a loading control, failed for two transiently arrested *pol32*Δ clones (clones c and d) implying disappearance of the primer(s) site from the VII-L terminus. Therefore, we verified the integrity of the VII-L terminus in *pol32*Δ clones by Southern blot and found that it was indeed rearranged in both clones in a similar manner, which is different from Y′ translocation (Figure S6). We failed to detect Y′ translocation in any of the *pol32*Δ clones that we have analyzed by Southern blot. The junction PCR product that is reduced but still detectable in *pol32*Δ clones could have been generated on single strand extension products that were terminated before reaching chromosome end. Thus, we concluded that Pol32-dependent BIR is essential for the completion of Y′ translocation.

To quantify the Y′ translocation efficiencies in the aforementioned deletion mutants, we employed real-time qPCR using two pairs of primers to amplify either the total amount of VII-L or the VII-L/Y′ junctions (Figure 5D). For this analysis we used DNA extracted from random clones isolated at ∼16 PD after Cre induction which were also analyzed by Southern blotting with VII-L-specific probe (Figure S7). We performed clonal analysis because it provides an unbiased snapshot of the repair frequencies at any given time, whereas bulk liquid cultures of telomerase-negative cells are strongly affected by selection for best-growing clones. We estimated that at the time of analysis (∼40 PD after Cre) on average 43.5% of VII-L ends in wild-type clones acquired Y′ element. The fraction of “repaired” VII-L was reduced to 18.2 and 6.6% in *rad51*Δ and *rad59*Δ, respectively, whereas it was at the background level in both *rad52*Δ and *pol32*Δ clones (Figure 5D). We inferred from these results that Rad52 predominantly cooperates with Rad59 to initiate Pol32-dependent BIR which leads to Y′ translocation. Nevertheless, Rad51 also appears to contribute in this process since it reduces the efficiency of Y′ translocations by at least two fold.

### Native telomeres shortened beyond critical length also acquire Y′ elements

To show that Y′ translocation is not a unique phenomenon that takes place at the modified VII-L end, but is common for eroded X-only telomeres, we used the same PCR approach to detect Y′ translocation on the native VI-R end (Figure S8A), which normally has only X element in the subtelomere region (W303 genome sequencing project, contig 00111). PCR product encompassing the junction between the VI-R end and the Y′ element was already detectable at 10 PD and increased further by 50 PD after telomerase inactivation (Figure S8B). Thus, erosion of the native telomeres in the absence of telomerase also leads to Y′ element translocations. Sequence analysis of the cloned PCR fragments confirmed that the VI-R/Y′ junction regions were predominantly amplified. However, we also identified a few clones resulted from mispriming on the X- and Y′-containing chromosomes V-R and XIII-L, which explains the background amplification before inactivation of telomerase.

**Figure 6:**
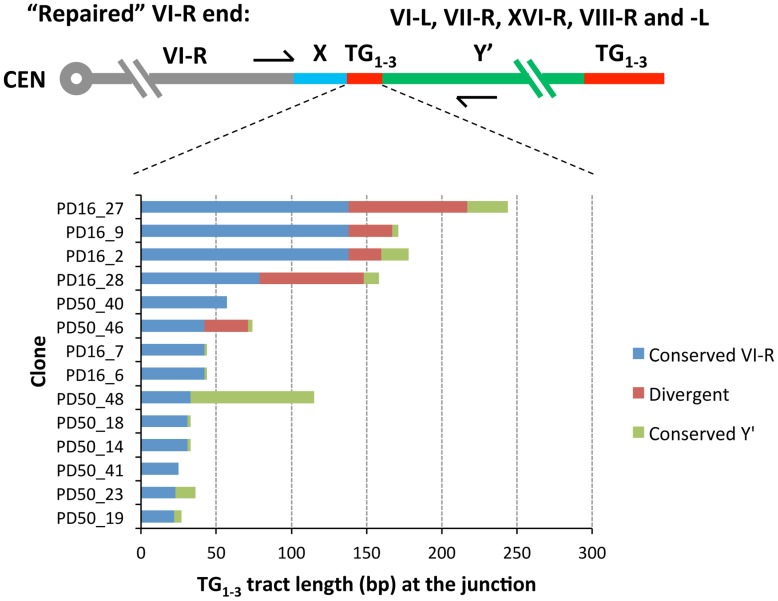
Sequence divergence within internal TG_1–3_ repeat tracts of the clones resulted from Y′ element translocation on the native VI-R end. DNA fragments encompassing VI-R/Y′ junctions were PCR-amplified from DNA of the “16 Rap1-bs” strain at either 16 or 50 PD after Cre induction, cloned and sequenced as described in Materials and Methods. The DNA sequences of the cloned VI-R/Y′ junctions were aligned at the VI-R side and the point of divergence was determined from sequence alignment. The analysis of recombination breakpoints was performed as described in the captions to Figure 4.

As expected, all clones contained TG_1–3_ repeat tracts at the junction between the VI-R end and Y′ element. Thus, similar to modified VII-L, native chromosome VI-R end also acquired Y′ element as a result of recombination between the eroded terminal and internal TG_1–3_ repeats. The extent of TG_1–3_ sequence identity at the VI-R side of the junction was limited to 60±45 (mean ±SD) nt (Figure 6), the minimum length of the native telomeres at the time they recombined. The divergent repeat sequence was shorter on average (or even absent) compared to that at the junction of modified VII-L (Figure 6). Notably, among the chromosome ends that served as Y′ donors we identified a greater variety of ends (Table 1), indicating that native telomere VI-R was less selective with respect to a Y′ donor. Of note, not all junctions between the X and Y′ elements contain perfect TG_1–3_ repeats, but they all tend to be G-rich and are all bound by Rap1 (Table1 and Figure S9).

### Rad59 promotes early acquisition of Y′ elements by native X-only telomeres

**Figure 7:**
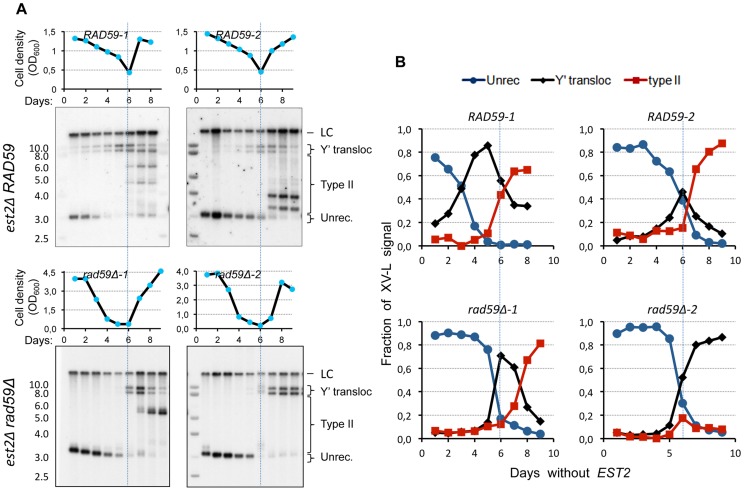
*RAD59* deletion results in a delay of Y′ translocation on the native X-only telomere XV-L. (A) Replicative senescence assays were performed in liquid culture by propagating the cells via serial dilutions to 1x10^5^ cells/ml every 24 h. The cell densities were estimated before each dilution. The DNA was digested with *Xho*I and subjected to Southern blot analysis with the XV-L subtelomere-specific probe, which also hybridizes to the internal subtelomeric fragment at III-R that serves as a loading control (LC). (B) Quantitation of the hybridization signal corresponding to different states of native telomere XV-L (as indicated in A).

Since the native VI-R/Y′ junctions showed simpler composition indicative of possibly greater efficiency of Y′ translocation, we attempted to detect them during the senescence time course by Southern blot directly in the bulk liquid cultures. To this end, we performed standard senescence assay in liquid with *est2*Δ spore clones. The samples were collected for DNA extraction daily at the time of culture dilution. *Xho*I-digested DNA was then Southern blotted and probed for the native X-only XV-L end using subtelomeric probe. We were surprised to find that two high-molecular-weight bands characteristic of Y′L and Y'S translocations were readily detectable early on during the outgrowth of the *est2*Δ cultures (Figure 7A, B; top panels). The same result was obtained with the native VI-R end (not shown). As expected, the timing of the onset of Y′ translocations correlated with the initial XV-L telomere length, which differed among the spore clones. The abundance of cells with Y′ translocated on XV-L in *est2*Δ cultures during presenescence was consistent with the notion of greater efficiency of the Y′ element translocation on the native X-only telomere than on the modified subtelomereless VII-L end.

We next asked whether the *RAD59* deletion, which reduced the frequency of Y′ translocation on the modified VII-L, would also compromise (or delay the onset of) Y′ translocations on the native XV-L as cells progress into senescence. Using the approach described above, we detected a substantial delay in the onset of Y′ translocations in the *est2 rad59*Δ relative to *est2*Δ clones (Figure 7). In the two representative *est2*Δ *rad59*Δ clones that are shown in Figure 7 (bottom panels), Y′ translocations were not detectable until the cells with very short XV-L telomere nearly disappeared from the population. In contrast, the cells with unrecombined short XV-L telomere and the cells which have already undergone Y′ translocation coexisted in the cultures of Rad59-proficient *est2*Δ clones long before they reached growth nadir. This result clearly points to the role of Rad59 in promoting Y′ translocation on the short native telomere. Note that in clone *rad59*Δ*-1*, type II pattern is seen after 8 days without telomerase. We usually observe that in liquid cultures, 75% of the clones are type I while 25% display a mixed pattern of type I and II (see Figure S11B).

### Rad59 delays the onset of crisis and promotes transition to type I at X-only telomeres

**Figure 8:**
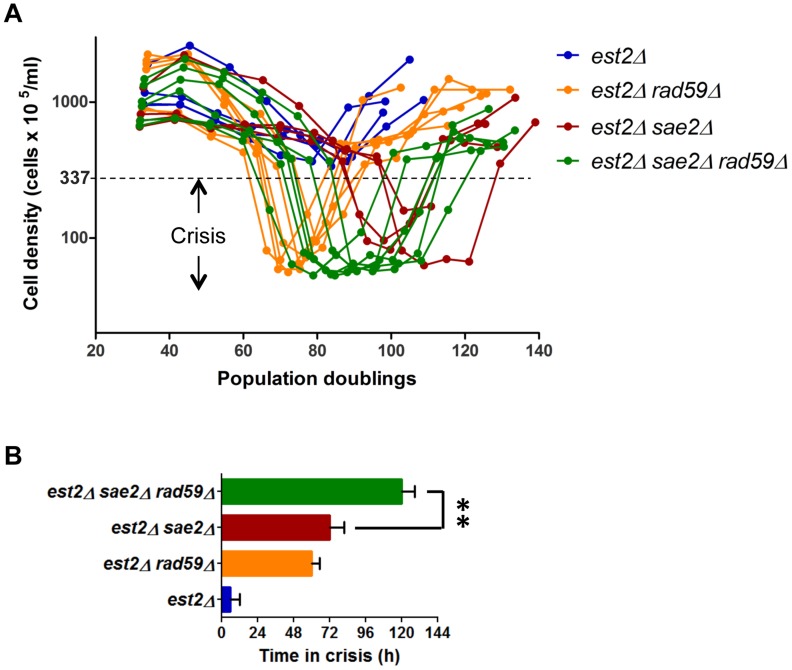
Rad59 sustains proliferation of cells approaching crisis and promotes type I survivors formation. (A) Replicative senescence assays were performed in liquid culture by propagating the cells via serial dilutions to 1x10^5^ cells/ml every 24 h. The cell densities were estimated before each dilution. (B) The crisis was arbitrary defined as the period when cell density did not exceed 3.37 x 10^7^ cells/ml (OD_600_≤1.0), which corresponds to ∼10 fold decline relative to initial measurement on day one. The bars represent mean time spent in crisis for multiple clones of each genotype assayed in (A), and the error bars are SEs. The significance of the difference between means was evaluated using unpaired two-tailed *t*-test (**, *P*<0.01).

The finding of short telomere repair by Y′ translocation raised a question whether this process can delay the onset of replicative senescence. Since Y′ element translocation is facilitated by Rad59 at X-only telomeres, we assayed the effect of *RAD59* deletion on the onset of proliferative decline in the absence of telomerase. To this end we performed standard senescence assays using multiple *est2Δ and est2Δ rad59Δ* clones. Clones lacking Rad59 lost proliferative capacity slightly earlier and exhibited fivefold lower cell densities at the nadir of growth (Figure 8A). This observation indicates that Rad59-facilitated repair of critically short telomeres contributes to sustain cell proliferation particularly when a population of telomerase-negative cells approaches growth nadir. Consistent with this notion, we found by ChIP-qPCR that Rad59 associates with telomeres during presenescence. Although Rad59 association varies considerably among the telomeres, it may peak abruptly (up to 25-fold increase) at certain X-only telomeres when culture approaches growth nadir (Figure S10A). On average, Rad59 association with terminal and internal (between X and Y′ elements) TG_1–3_ repeats increases many fold as telomerase-negative cells progress into senescence (Figure S10B), highlighting the role of Rad59 in the conversion of X-only into Y′ telomeres.

We next reasoned that Y′ element translocation could be the initial step of type I telomere pattern formation at X-only telomeres. This premise seemingly contradicts established genetic requirements for survivor formation since type I survivors are predominantly obtained in cells lacking Rad59 [12], [13], [29]. The latter is due to the fact that Rad59 deletion greatly impedes type II. However, this does not exclude a possibility that Rad59 also facilitates transition to type I, particularly at X-only telomeres, although it is not strictly required. To reveal such a role of Rad59, we had to prevent the dominant type II pathway. This was performed by deleting *SAE2* whose deletion strongly inhibits type II formation (Figure S11). We therefore compared the efficiency of type I survivor formation in *est2Δ sae2Δ* and *est2Δ sae2Δ rad59Δ* mutants (Figure 8B). We found that the triple mutant clones spent longer time in crisis suggesting reduced efficiency of type I survivor formation.

### Y′ element acquisition and its amplification have distinct genetic requirements

**Figure 9:**
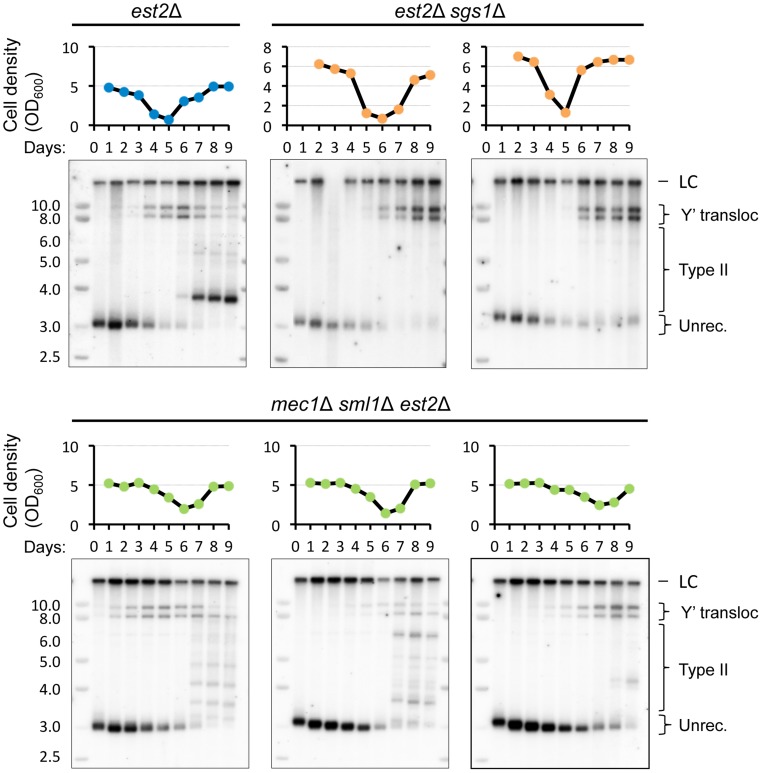
*SGS1* but not *MEC1* deletion delays Y′ translocation on the native telomere XV-L. Southern blot analysis of the Y′ translocation kinetics on native telomere XV-L during senescence of the telomerase-negative strains with indicated gene deletions. Refer to the caption to Figure 7 for details of the replicative senescence assay and Southern blot analysis.

The involvement of *RAD59* in Y′ translocation raised a question whether the other genes of the type II survivor pathway may also contribute to Y′ acquisition. To this end, we compared the kinetics of Y′ acquisition by the native telomere XV-L between *est2Δ* and *sgs1Δ est2Δ* cells. We chose to delete *SGS1*, another gene required for type II survivors to arise [13], [15], [16], [19], because unlike the genes encoding the subunits of MRX complex it does not cause short telomeres, which would complicate comparative analysis of Y′ translocation kinetics since short telomeres acquire Y′ faster. We found that although *est2Δ* and *sgs1Δ est2Δ* clones started to senesce with XV-L telomere of comparable size, there was a substantial delay in Y′ translocation on XV-L in *sgs1Δ est2Δ* compared to *est2Δ* cells (Figure 9). Therefore, Sgs1 also appear to promote Y′ element acquisition. As expected, XV-L telomere converted to type I pattern by the time *sgs1Δ est2Δ* cells generated survivors. Remarkably, recombination between Y′ elements increases in *sgs1Δ* mutants [39], thus the inhibitory effect of *SGS1* deletion on Y′ translocation further highlights the mechanistic difference between largely *RAD51*-independent Y′ acquisition and *RAD51*-dependent Y′ amplification, the two steps of type I survivor formation.

Since we found that short telomere repair is associated with transient arrest, we asked whether the checkpoint function is required for Y′ translocation. To address it, we deleted *MEC1*, which is required for G2/M arrest in telomerase-deficient cells [40] and also mediates type II recombination [41]. As expected, *mec1Δ sml1Δ* mutant clones exhibited flatter senescence profiles indicative of defective cell cycle arrest in response to telomere shortening and delayed senescence as reported previously [8], and yet Y′ translocation was not affected in any of the three clones analyzed (Figure 9). We concluded that checkpoint function is not required for Y′ acquisition by X-only telomeres. This is in contrast to deleting *RAD59* and *SGS1*, which both substantially delay Y′ acquisition.

## Discussion

Many studies from several laboratories have characterized the genetic pathways contributing to survivor formation [42]. In this study we demonstrated that a single telomere that was experimentally shortened in the absence of telomerase can acquire during presenescence Y′ element along with the terminal TG_1–3_ repeats from other chromosome ends which terminal repeats are still sufficiently long. Sequencing of the Y′ translocation junctions clearly evidenced that Y′ translocation was initiated by recombination between the short terminal TG_1–3_ repeats of the TelVII-L and the internal TG_1–3_ tracts located between the X and Y′ elements on certain native chromosome ends thereby confirming the model of Lundblad and Blackburn [10]. In addition, our results suggest that there is a continuous repair of the shortest telomeres that delays erosion into the subtelomere and allow the cell to escape DNA damage checkpoint activation. We showed that Y′ translocation was entirely *RAD52*-dependent, and was further promoted by *RAD59*. In contrast, *RAD51* deletion had rather modest effect on the efficiency of Y′ translocation (two fold reduction), and Y′ translocations on VII-L end were detectable in *rad51*Δ clones. Therefore, Rad51-ssDNA filament formation and strand invasion do not seem to be obligatory for TG_1–3_ repeat recombination; instead, it could be accomplished by Rad52 strand annealing activity that is stimulated by Rad59. This mechanism bears strong similarities with the “short tract homology” recombination that has been described in the other context [43]. It remains possible that Rad51 affects Y′ translocations indirectly via its involvement in the recombination among the Y′ elements themselves [44]. Another question that rises with respect to Rad51-independent recombination between TG_1–3_ repeats is how the G-strand overhang anneals to the internal TG_1–3_ repeats, which are normally double-stranded. Possibly, this single-strand annealing occurs during subtelomere replication when the single strand regions are exposed [45], particularly when the replication forks are posed at the internal TG_1–3_ sequences [46].

In this study, we showed that such recombination events also occurred at the native telomeres VI-R and XV-L indicating that these rearrangements are physiological in nature. We found that modified subtelomereless VII-L and native X-only telomeres behave surprisingly different with respect to the efficiency of Y′ acquisition. Low efficiency of Y′ translocation on the modified VII-L end might be explained by its poor clustering with the other telomeres due to absence of subtelomeric elements. Indeed, intranuclear localization of the truncated TelVII-L is insensitive to the absence of either Sir3 or Yku [47], whereas these proteins participate in targeting native telomeres to the nuclear periphery [48], [49]. Poor clustering and failure to localize to nuclear compartments which favour recombination could be responsible for low efficiency of Y′ translocation and may explain why single experimentally shortened telomere accelerates senescence [8], [28].

Our results suggest that the Rad59-facilitated recombination between the terminal and internal TG_1–3_ repeats could be responsible for initial spreading of certain Y′ elements on X-only telomeres. We demonstrated that disappearance of the bands corresponding to X-only telomeres from Southern blots as telomerase-negative cultures progress into crisis is indeed a consequence of Y′ element acquisition that is promoted by Rad59. One can, therefore, envisage that the clones that arise via Y′ translocations are the precursors of type I survivors. The homogenization of the subtelomeric sequences due to preferential translocation of a certain class of Y′ elements can further promote perhaps more efficient Rad51-dependent BIR initiated within Y′ elements resulting in their amplification that is observed in type I survivors [10]. Remarkably, the strains that harbour Y′ elements at all chromosome ends due to previous history as a telomerase-defective survivor form survivors more readily when rendered telomerase-negative again [50]. This observation suggests that spreading of the Y′ elements to all ends could be one factor that limits the rate of type I survivor formation. Consistent with this proposition, we found that Rad59 accelerates type I survivor formation when type II survivor pathway is inhibited.

The initial step of Y′ acquisition involves heteroduplex formation by TG_1–3_ repeats that contain mismatches and is likely recognized by mismatch-repair proteins [51], [52]. It is therefore possible that the homeologous heteroduplexes formed during Rad59-dependent single-strand annealing are often rejected, and this may lead to reiterative rounds of repair synthesis which could effectively provide a way of TG_1–3_ tract expansion in the absence of telomerase. Indeed, we have identified a few clones with substantially long TG_1–3_ tracts at the breakpoint of recombination, which could have resulted from reiterative repair synthesis. We could speculate that once very long internal tracts are generated, they can be excised as circles via intramolecular recombination events (perhaps in a few steps) [14], [53]. The ultimate escape mechanism would be achieved via conversion to type II survivors, which are thought to amplify their terminal repeats via rolling circle replication. This could explain the apparent contradiction between the fact that circular DNA molecules are efficiently generated only when telomeric repeat tracts are abnormally elongated [54] and that only short telomeres engage in type II recombination [14].

Interestingly, we found that Rap1 was strongly enriched at the internal TG_1–3_ tracts located between the X and Y′ elements of the donor telomeres (Figure S9 and Table 1, see also [55]). Rap1 at these internal TG_1–3_ tracts was not relocalized upon critical shortening of telomeres [55]. It is conceivable that these internal Rap1 binding sites are also bound by the Sir proteins [56]. Notably, there is no homology between the modified TelVII-L and the Y′ donor chromosome end outside of the TG_1–3_ repeat tracts. Nevertheless, the apparent efficiency of Rad51-independent single strand annealing that relies exclusively on short homeologous TG_1–3_ repeat sequences is rather high, which might be due to spatial clustering of the telomeres in the yeast nucleus [57]. Indeed, for double-strand break repair, it has been recently shown that proximity of the donor sequence promotes homologous recombination [58]. The choice of the Y′ donor does not appear random. Whether this preference reflects the proximity between the chromosome ends in the nucleus or is influenced by other factors is currently unknown. Of note, among all chromosome ends, the VI-L end harbours one of the longest (139 bp-long) internal TG_1–3_ tract between the X and Y′ elements; and VII-R end is located at approximately the same distance from the centromere as the experimental VII-L end on the opposite arm of the same chromosome, which is consistent with Rabl configuration [59]. It has been also reported that Rap1 had the intrinsic ability to locally distort telomeric double-stranded DNA provoking local conformational changes characteristic of single strand [60]. Therefore, subtelomeric Rap1 binding could favour homologous recombination by creating local structures that are amenable to annealing with single-stranded overhang. In addition, Rap1 contains four putative SIMs, and thus, it may potentially recruit SUMOylated Rad52 and Rad59 to TG_1–3_ tracts.

Although our study focuses on short telomere repair by Y′ translocation, the BIR events leading to only terminal TG_1–3_ tract extension are also possible in telomerase-negative yeast. These events are readily detectable by Southern blot in survivors [14], [22] since they often result in large increase of terminal TG_1–3_ tract length, but this is not the case in pre-senescent cells. Direct sequencing of the terminal repeats showed that they do occasionally get extended in pre-senescent cells, although there is a controversy of whether the short or long tracts are preferentially extended [21], [22]. In any case, recombination between terminal repeats cannot indefinitely sustain proliferation of telomerase-negative cells, whereas Y′ translocation leads toward type I telomere formation, which likely requires additional changes such as chromatin structure alteration [19].

In summary, we have characterized a repair mechanism that acts upon short X-only telomeres in budding yeast lacking telomerase during presenescence. This repair mechanism does not require Rad51 and depends on annealing between short homeologous sequences which is stimulated by Rad59. Unlike a single unrepairable DSB, a single critically short telomere is not immediately lethal for a cell in the absence of telomerase as long as there is a reserve of long telomeres on other chromosome ends. Remarkably, Rad51 independence of the short telomere rescue pathway points to yet another problem caused by telomerase inactivation which resolution depends on Rad51-dependent HR, since deletion of *RAD51* is known to cause early loss of viability of telomerase-negative cells.

## Materials and Methods

### Strains and plasmids

All yeast strains used in this study were from the W303 background (see genotypes in Table S2). Strains used to analyze telomere rearrangement in the absence of telomerase were derivatives of the YAB892 and YAB893, which have 0 and 16 Rap1-binding sites, respectively, flanked by loxP sites at the modified telomere VII-L [31]. To obtain Tet-Off *TLC1* derivatives, these strains were crossed to the *tetO_2_*-*TLC1* strain. The inducible *EST2* deletion derivatives (double Cre-loxP) were generated by first transforming the cells with the pDS381 plasmid [33] carrying *EST2* gene linked to the *ADE2* marker and the loxP-flanked *ARS/CEN* region, and then replacing the endogenous *EST2* locus with *KAN* ^r^ cassette. All other gene disruptions were carried out by PCR-based methods resulting in the replacement of targeted loci with the *TRP1* marker.

### Inducible shortening of the TelVII-L and inactivation of telomerase

To induce genome-integrated *GAL*p-*CRE* for simultaneous TelVII-L shortening and loss of the plasmid-borne *EST2*, overnight cultures growing in SC medium lacking lysine and adenine and containing 2% raffinose were diluted (1∶20) into complete YEP medium containing 2% galactose. After 24 hours of induction the cells were diluted into YPD to prevent genome damage by Cre at non-specific sites. To inhibit telomerase in the Tet-off *TLC1* strains, the cultures grown in SC medium lacking lysine and containing 2% raffinose were supplemented with doxycycline (10 µg/µl), 12 hours before induction of TelVII-L shortening by switching cells to galactose for the next 24 hours as described above. The doxycycline concentration was maintained constant throughout the experiments in all media.

### Microcolony assay and isolation of the clones with rearranged VII-L end

To determine the ability of individual telomerase-negative cells to form microcolonies, the double Cre-loxP cells were micromanipulated onto a grid of YPD agar at 36 h after induction of Cre with galactose in liquid culture. Alternatively, the Tet-off *TLC1* derivatives were micromanipulated onto the YPD agar freshly supplemented with Dox (20 µg/µl) at 24 h after Cre induction and 48 h after repression of *TLC1* with Dox. The microcolony formation was monitored by counting the number of cells in each grid position at 2, 4, and 8 h after micromanipulation. The plates were incubated for additional 4 days to allow formation of visible colonies. The colonies which exhibited growth delay at the time or soon after micromanipulation were chosen for VII-L end analysis. In addition, the colonies with deeply nibbled edges and typically smaller size were included regardless of the growth delay. The clones that exhibited robust expansion and formed large colonies with smooth edges were used as controls.

### Southern blot analysis of the VII-L and bulk telomeres

To determine the state of the VII-L end before and after induction of Cre expression, genomic DNA was digested simultaneously with *Mfe*I and *Pac*I. The resulting fragments were separated by 0.9% agarose gel electrophoresis, transferred on Hybond N+, and hybridized with ^32^P-labeled 252 bp-long probe that was generated by PCR (see Table S1 for primers sequences) using YAB892 genomic DNA as a template. The rearrangement of the VII-L end in clonal populations recovered from transient arrest was analyzed similarly, except for two aliquots of DNA were digested separately with either *Mfe*I or *Pac*I. To determine the length of native telomeres, *Xho*I-digested yeast DNA was subjected to 0.8% agarose gel electrophoresis and hybridized with a ^32^P-labeled (TG_1–3_)_n_ probe. The probes were labeled by random priming using Klenow fragment exo-, and all hybridizations were performed in Church buffer at 58°C.

### PCR across the translocation junction

To amplify the regions encompassing putative Y′ translocation junctions at either modified VII-L or native VI-R ends, the chromosome end-specific primers were designed to anneal ∼500 bp away from terminal TG_1–3_ repeats, and the Y′ element-specific primer sites were chosen in the centromere-proximal region that is conserved among all Y′ elements (Table S1). The Y′ sequence alignments are available from Ed Louis at http://www2.le.ac.uk/colleges/medbiopsych/research/gact/resources/yeast-telomeres. Analytical PCR was performed using Phusion DNA Polymerase (Thermo Scientific) in 1xHF buffer and 1 ng/µl genomic DNA purified via phenol-chlorophorm extraction. Primers were used at 500 nM, and the annealing temperature was set at 3°C above the T_m_ of the least stable primer.

### Cloning and sequencing of the translocation junction regions

For cloning, PCR across the junction was performed using Taq DNA polymerase to produce fragments with 3′ A overhangs, and the purified product was inserted into the pCR2.1 vector via one-step TA cloning (Invitrogen). The inserts were sequenced from both ends using M13F-20 and M13-26REV primers by Sanger method (at Beckman Coulter). As a rule, the read going through the TG-rich strand failed at the junction, so the entire sequence was assembled from both reads.

### Rad59 ChIP-qPCR

Rad59 was 13xMyc epitope–tagged at the C-terminus using one-step PCR. ChIP was performed as previously described [61]. Briefly, chromatin was cross-linked with 1% formaldehyde and sonicated to an average 200- to 500-bp DNA fragment size. After clarifying centrifugation, soluble chromatin was incubated with mouse anti-Myc tag monoclonal antibodies (9E10) and immunocomplexes were bound to magnetic Dynabeads Protein G (Novex). Following successive washes in standard solutions, Rad59-Myc bound chromatin was eluted from beads and incubated at 68°C to reverse crosslinks. DNA purified from the immunoprecipitates and inputs was quantified by real-time qPCR using chromosome end-specific primers listed in Table S1. The enrichment of the telomere-specific sequences bound by Rad59 was normalized to input and an unaffected *GAL2* locus.

### Rap1 ChIP-on-chip

Rap1 ChIP experiments (duplicates) were performed with anti-Rap1 antibodies kindly provided by David Shore (University of Geneva). Rap1 ChIP and input DNA samples were hybridized to Nimblegen S. cerevisiae high density tiling arrays that were designed by us in collaboration with Frédéric Devaux (Ecole Normale de Paris) and Nimblegen to cover the entire genome. They contain 50 nt-long oligonucleotides separated by ∼15 nt-long gaps. The chip covers both strands of *S. cerevisiae* genome. Hybridizations and data analysis were performed by Nimblegen (Roche NimbleGen). Rap1 peaks were visualized with the signalMap software (Nimblegen) or with the Integrative Genomics Viewer (Broad Institute). The genome-wide Rap1 binding profiles were consistent with the recent published studies [55].

## Supporting Information

Figure S1 Apparent limit of the terminal TG_1–3_ repeat tract shortening observed in liquid culture. The VII-L telomere length distribution shifts in the “0” and “16 Rap1-bs” Cre-loxP strains after *EST2* deletion. The signal intensities were quantified from the Southern blots shown in Figure 1B using ImageQuant 5.2 (Molecular Dynamics). Each lane was divided into 50 even intervals and the volumes adjusted for background were plotted against the mean TRF size (bp) for each interval. The lengths of the TG_1–3_ repeat tracts were calculated by subtracting the non-telomeric portion of the VII-L *Pac*I TRF (288 bp) from the mean TRF size (bp) of each interval.
(DOCX)

Figure S2 Southern blot analysis of the TelVII-L state in the randomly chosen “0” and “16Rap1-bs” clones isolated at 12 and 18 PD after Cre induction. (A) DNA was digested with *Pac*I+*Mfe*I and hybridized with VII-L-specific probe. Lanes labeled “Bulk” contain DNA extracted from bulk liquid cultures at the time when clones were isolated. Symbols marking the lanes are explained in the footnotes. In the bottom right panel, the blue bars across the lanes indicate groups of subclones marked A–D, which were obtained by sequential micromanipulation of the cells that came out of the arrest. Note that a fuzzy band migrating just below 3 kb, partially overlapping with VII-L/Y'S fragments, is most likely resected VII-L terminal fragment which is largely single-stranded. (B) Schematic of the VII-L end showing probe annealing site and expected sizes of the restriction fragments visualized on Southern blots before and after Y′ element translocation. (C) Contingency tables showing the frequency of Y′ translocation for the “arrested” and “non-arrested” groups of “0 and 16Rap1-bs” clones each isolated at 12 and 18 PD after Cre induction. The groups of “16Rap1-bs” subclones, labeled A–D in (A) have been counted as one clone isolated at 18 PD.
(DOCX)

Figure S3 Sequence analysis of the recombination breakpoint within internal TG_1–3_ tract. (A) The extent of identical TG_1–3_ repeat sequences in the VII-L/Y′ junction clones. DNA fragments encompassing VII-L/Y′ junctions were PCR-amplified from DNA of “16 Rap1-bs” strain at either 10 or 50 PD after Cre induction, cloned and sequenced. The TG_1–3_ repeat sequences present in the cloned VII-L/Y′ junctions were aligned at the *Bam*HI site (the end of modified VII-L). The repeats past the point of divergence were removed to expose the extent of sequence identity at the VII-L end. The sequences were aligned with ClustalW and the resulted alignments were manually curated and shaded in BioEdit. (B) An example of a simple recombination breakpoint within internal TG_1–3_ tract. The terminal sequence of the experimentally shortened telomere VII-L inferred from the alignment of multiple junction clones in marked with blue. The newly synthesized repeats are in red. The 139 bp-long sequence of the internal TG_1–3_ repeats of the chromosome VI-L which served as Y′ donor is in black. The heteroduplex formed between the terminal and internal repeats was 26 bp-long and contained two mismatches. Importantly, neither 5′ end resection nor D-loop branch migration are expected to extend the heteroduplex length since artificial VII-L end does not share homology with other chromosome ends outside TG_1–3_ repeats. Therefore, Y′ translocation depends exclusively on homeologous pairing between the short tracts of TG_1–3_ repeats.
(DOCX)

Figure S4 The effect of *TLC1* repression on VII-L (top panels) and bulk (bottom panels) telomere length. Wild-type and Tet-off *TLC1* cells with modified TelVII-L (16 Rap1-bs) (see Figure 1A) were grown in S-raffinose –Lys in the presence of Dox for 24 h and then shifted to galactose for the next 24 h to induce Cre expression. Consequently, the cultures were propagated by serial dilutions in YPD in the presence of Dox. DNA extracted from the samples taken at indicated PDs after Cre induction was digested with either *Pac*I and *Mfe*I or *Xho*I and subjected to Southern blot analyses with either VII-L-specific (see Figure 1A) or TG_1–3_ probe to visualize VII-L and bulk telomeres, respectively. The pattern of telomeres observed in the Tet-off *TLC1* strain during the last four time points is characteristic for type II survivors.
(DOCX)

Figure S5 Survival of the telomerase-inhibited clones with indicated gene deletions. Tet-off *TLC1* strains with indicated gene deletions were grown in the presence of Dox to suppress *TLC1* expression. Abrupt shortening of the TelVII-L in “16 Rap1-bs” strain was induced via transient induction of pGAL-Cre by shifting cells to galactose for 24 h. At the end of Cre induction, single cells were micromanipulated on a grid on YPD agar supplemented with Dox. Cell divisions were monitored microscopically and the numbers of cells in microcolonies were counted at 4 and 6 h after plating. Images of the plates taken 3 days after single cells were micromanipulated are shown. Survival graph shows total survival (all micromanipulated cells, blue bars) and survival after arrest (arrested within 8 h after plating, pink bars).
(DOCX)

Figure S6 Southern blot analysis of the VII-L end in *pol32Δ* clones. DNA extracted from bulk, B, and clonal, a–f, populations (see caption to Figure 5 for details of the experiment) was digested separately with either *Pac*I or *Mfe*I (restriction sites positions at the VII-L end are shown in the diagram of Figure 2C). Digested DNA was subjected to Southern blot analyses with VII-L-specific probe. The brackets indicate terminal fragments of the telomere VII-L, whereas open arrowheads point to the fragment resulted from VII-L end rearrangement.
(DOCX)

Figure S7 **S**outhern blot analysis of the TelVII-L state in the randomly chosen *rad59*Δ and *rad51*Δ “16Rap1-bs” clones isolated at ∼16 PD after Cre induction. See legend to figure S2 for further details.
(DOCX)

Figure S8 Y′ element translocation on the native telomere VI-R can also be detected by PCR across the junction. (A) Schematic of the primer design for detection of the Y′ translocation onto native VI-R end, which contains only X element. Y′ donor refers to any X and Y′ element containing chromosome end. (B) Semi-quantitative PCR across the VI-R/Y′ junction. DNA was extracted from 2x Cre-loxP strains grown in liquid culture at 0, 10, 50 PD after induction of Cre expression (as in Figures 1B and 3B) and used as a template in PCR reaction with two different pairs of primers designed to amplify the VI-R/Y′ junction. The bracket indicates expected size range of the junction PCR product. TC – template control, a 0.47-kb PCR product amplified at the very terminus of native VI-R end.
(DOCX)

Figure S9 Rap1 binds the sequences between X and Y′ and between tandem Y′ elements. Rap1 ChIP tiling array data are shown for the terminal 12.5 kb of the selected chromosome ends with the following subtelomere organization: I-L, X element only; II-L, short Y′ and X elements are separated by degenerate TG_1–3_ repeats; VI-L, short Y′ and X elements are separated by 139 bp of TG_1–3_ repeats; two short tandem Y′ elements are separated by 64 bp of TG_1–3_ repeats, and the proximal Y′ and X elements are separated by 163 bp of TG_1–3_ repeats; XIV-L, long Y′ and X elements are separated by degenerate TG_1–3_ repeats. The G-rich sequences separating subtelomeric elements were considered degenerate if there were no stretches of TG_1–3_ longer than 12 bp. Data are plotted for a set range (−0.80–8.40) for all chromosome ends using IGV version 2.3.25.
(DOCX)

Figure S10 Rad59 association with TG_1–3_ repeats during proliferative decline of *est2Δ* cells. Rad59 binding to terminal (telomeric) and internal (subtelomeric) TG_1–3_ repeats was determined by ChIP-qPCR during outgrowth of the *Rad59-13Myc est2Δ* spore clone up to the peak of crisis (maximum decline of the doubling time). Enrichment of the telomere-specific sequences immunoprecipitated with Rad59 was determined relative to non-specific locus as follows: fold enrichment  =  (Telo_ChIP_/Telo_Input_)/(*GAL2*_ChIP_/*GAL2*_Input_). (A) Rad59 ChIP at individual X-only telomeres. (B) Rad59 ChIP at an average X-only telomere (mean ±SE, n = 4) and at the internal sequences between X and Y′ elements.
(DOCX)

Figure S11 *SAE2* deletion substantially reduces the efficiency of type II survivor formation. (A) *est2Δ sae2Δ* mutants generate predominantly type I survivors, as opposed to *est2Δ*, which generate mostly type II survivors in liquid culture. Only the clones that survived via type I pathway were included in the comparison with triple *est2Δ sae2Δ rad59Δ* mutants (see Figure 8A and 8B), which generate exclusively type I survivors. DNA was extracted at the indicated days during senescence time course, digested with *Xho*I, subjected to Southern blot, and probed for TG_1–3_ repeats. The graphs above the Southern blots show the growth profiles during senescence in liquid cultures for indicated genotypes. (B) Histogram showing the fractions of different survivor types generated by the indicated genotypes.
(DOCX)

Table S1 Primers used in this study.
(DOCX)

Table S2 Yeast strains used in this study.
(DOCX)
