# Supplementary material for: Rad59-Facilitated Acquisition of Y′ Elements by Short Telomeres Delays the Onset of Senescence
Source: PLoS Genet. 2014 Nov 6;10(11):e1004736. doi: 10.1371/journal.pgen.1004736 (PMC4222662; doi:10.1371/journal.pgen.1004736)
Supplement: Figure S1 — Apparent limit of the terminal TG1–3 repeat tract shortening observed in liquid culture. The VII-L telomere length distribution shifts in the “0” and “16 Rap1-bs” Cre-loxP strains after EST2 deletion. The signal intensities were quantified from the Southern blots shown in Figure 1B using ImageQuant 5.2 (Molecular Dynamics). Each lane was divided into 50 even intervals and the volumes adjusted for background were plotted against the mean TRF size (bp) for each interval. The lengths of the TG1–3 repeat tracts were calculated by subtracting the non-telomeric portion of the VII-L PacI TRF (288 bp) from the mean TRF size (bp) of each interval. (DOCX) [file pgen.1004736.s001.docx]

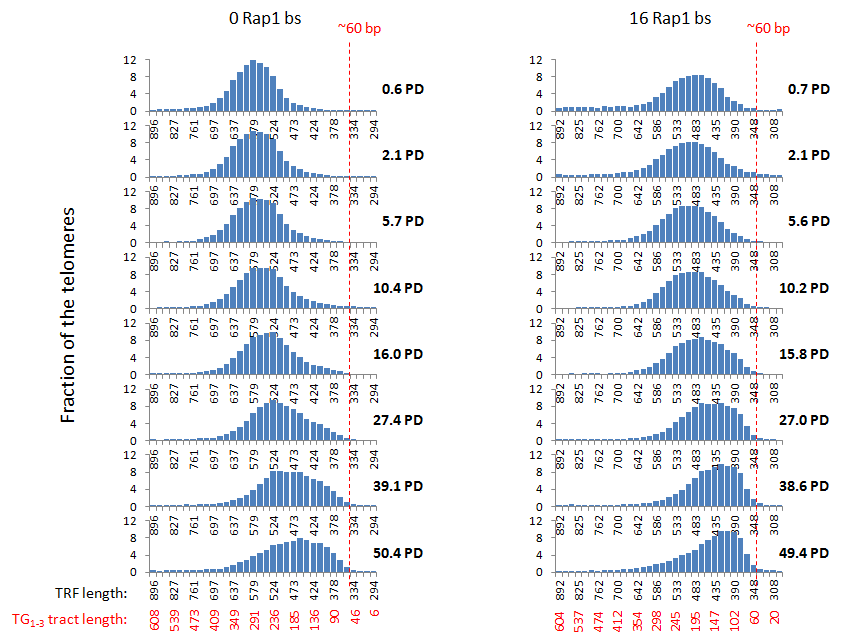


**Figure S1. Apparent limit of the terminal TG_1-3_ repeat tract shortening observed in liquid culture.** The VII-L telomere length distribution shifts in the “0” and “16 Rap1-bs” Cre-loxP strains after *EST2* deletion. The signal intensities were quantified from the Southern blots shown in Figure 1B using ImageQuant 5.2 (Molecular Dynamics). Each lane was divided into 50 even intervals and the volumes adjusted for background were plotted against the mean TRF size (bp) for each interval. The lengths of the TG_1-3_ repeat tracts were calculated by subtracting the non-telomeric portion of the VII-L *Pac*I TRF (288 bp) from the mean TRF size (bp) of each interval.
