# Supplementary material for: Rad59-Facilitated Acquisition of Y′ Elements by Short Telomeres Delays the Onset of Senescence
Source: PLoS Genet. 2014 Nov 6;10(11):e1004736. doi: 10.1371/journal.pgen.1004736 (PMC4222662; doi:10.1371/journal.pgen.1004736)
Supplement: Figure S2 — Southern blot analysis of the TelVII-L state in the randomly chosen “0” and “16Rap1-bs” clones isolated at 12 and 18 PD after Cre induction. (A) DNA was digested with PacI+MfeI and hybridized with VII-L-specific probe. Lanes labeled “Bulk” contain DNA extracted from bulk liquid cultures at the time when clones were isolated. Symbols marking the lanes are explained in the footnotes. In the bottom right panel, the blue bars across the lanes indicate groups of subclones marked A–D, which were obtained by sequential micromanipulation of the cells that came out of the arrest. Note that a fuzzy band migrating just below 3 kb, partially overlapping with VII-L/Y'S fragments, is most likely resected VII-L terminal fragment which is largely single-stranded. (B) Schematic of the VII-L end showing probe annealing site and expected sizes of the restriction fragments visualized on Southern blots before and after Y′ element translocation. (C) Contingency tables showing the frequency of Y′ translocation for the “arrested” and “non-arrested” groups of “0 and 16Rap1-bs” clones each isolated at 12 and 18 PD after Cre induction. The groups of “16Rap1-bs” subclones, labeled A–D in (A) have been counted as one clone isolated at 18 PD. (DOCX) [file pgen.1004736.s002.docx]

**A**


**Figure S2.** **Southern blot analysis of the TelVII-L state in the randomly chosen “0” and “16Rap1-bs” clones isolated at 12 and 18 PD after Cre induction.** (A) DNA was digested with *Pac*I+*Mfe*I and hybridized with VII-L-specific probe. Lanes labeled “Bulk” contain DNA extracted from bulk liquid cultures at the time when clones were isolated. Symbols marking the lanes are explained in the footnotes. In the bottom right panel, the blue bars across the lanes indicate groups of subclones marked A-D, which were obtained by sequential micromanipulation of the cells that came out of the arrest. Note that a fuzzy band migrating just below 3 kb, partially overlapping with VII-L/Y’S fragments, is most likely resected VII-L terminal fragment which is largely single-stranded. (B) Schematic of the VII-L end showing probe annealing site and expected sizes of the restriction fragments visualized on Southern blots before and after Y’ element translocation. (C) Contingency tables showing the frequency of Y’ translocation for the “arrested” and “non-arrested” groups of “0 and 16Rap1-bs” clones each isolated at 12 and 18 PD after Cre induction. The groups of “16Rap1-bs” subclones, labeled A-D in (A) have been counted as one clone isolated at 18 PD.
