# Supplementary material for: Rad59-Facilitated Acquisition of Y′ Elements by Short Telomeres Delays the Onset of Senescence
Source: PLoS Genet. 2014 Nov 6;10(11):e1004736. doi: 10.1371/journal.pgen.1004736 (PMC4222662; doi:10.1371/journal.pgen.1004736)
Supplement: Figure S3 — Sequence analysis of the recombination breakpoint within internal TG1–3 tract. (A) The extent of identical TG1–3 repeat sequences in the VII-L/Y′ junction clones. DNA fragments encompassing VII-L/Y′ junctions were PCR-amplified from DNA of “16 Rap1-bs” strain at either 10 or 50 PD after Cre induction, cloned and sequenced. The TG1–3 repeat sequences present in the cloned VII-L/Y′ junctions were aligned at the BamHI site (the end of modified VII-L). The repeats past the point of divergence were removed to expose the extent of sequence identity at the VII-L end. The sequences were aligned with ClustalW and the resulted alignments were manually curated and shaded in BioEdit. (B) An example of a simple recombination breakpoint within internal TG1–3 tract. The terminal sequence of the experimentally shortened telomere VII-L inferred from the alignment of multiple junction clones in marked with blue. The newly synthesized repeats are in red. The 139 bp-long sequence of the internal TG1–3 repeats of the chromosome VI-L which served as Y′ donor is in black. The heteroduplex formed between the terminal and internal repeats was 26 bp-long and contained two mismatches. Importantly, neither 5′ end resection nor D-loop branch migration are expected to extend the heteroduplex length since artificial VII-L end does not share homology with other chromosome ends outside TG1–3 repeats. Therefore, Y′ translocation depends exclusively on homeologous pairing between the short tracts of TG1–3 repeats. (DOCX) [file pgen.1004736.s003.docx]

**A**

**B**

Clone_H7 1 TGTGGTGTGTGGGTGTGGGTGTGGTGTGTGGGTGTGGGTGTGTGGGTGTGGTGTGTGTGG 60

||||| |||| |||||||||||||||||||||||||||||||||||||||||||||||||

VI-L 72 TGTGG-GTGT-GGTGTGGGTGTGGTGTGTGGGTGTGGGTGTGTGGGTGTGGTGTGTGTGG 129

Clone_H7 61 GTGTGTGGGT 70 -> Y’ element

||||||||||

VI-L 130 GTGTGTGGGT 139-> Y’ element

**Figure S3. Sequence analysis of the recombination breakpoint within internal TG_1-3_ tract.** (A) The extent of identical TG_1-3_ repeat sequences in the VII-L/Y’ junction clones. DNA fragments encompassing VII-L/Y’ junctions were PCR-amplified from DNA of “16 Rap1-bs” strain at either 10 or 50 PD after Cre induction, cloned and sequenced. The TG_1-3_ repeat sequences present in the cloned VII-L/Y’ junctions were aligned at the *Bam*HI site (the end of modified VII-L). The repeats past the point of divergence were removed to expose the extent of sequence identity at the VII-L end. The sequences were aligned with ClustalW and the resulted alignments were manually curated and shaded in BioEdit. (B) An example of a simple recombination breakpoint within internal TG_1-3_ tract. The terminal sequence of the experimentally shortened telomere VII-L inferred from the alignment of multiple junction clones in marked with blue. The newly synthesized repeats are in red. The 139 bp-long sequence of the internal TG_1-3_ repeats of the chromosome VI-L which served as Y’ donor is in black. The heteroduplex formed between the terminal and internal repeats was 26 bp-long and contained two mismatches. Importantly, neither 5’ end resection nor D-loop branch migration are expected to extend the heteroduplex length since artificial VII-L end does not share homology with other chromosome ends outside TG_1-3_ repeats. Therefore, Y’ translocation depends exclusively on homeologous pairing between the short tracts of TG_1-3_ repeats.
