# Supplementary material for: Rad59-Facilitated Acquisition of Y′ Elements by Short Telomeres Delays the Onset of Senescence
Source: PLoS Genet. 2014 Nov 6;10(11):e1004736. doi: 10.1371/journal.pgen.1004736 (PMC4222662; doi:10.1371/journal.pgen.1004736)
Supplement: Figure S4 — The effect of TLC1 repression on VII-L (top panels) and bulk (bottom panels) telomere length. Wild-type and Tet-off TLC1 cells with modified TelVII-L (16 Rap1-bs) (see Figure 1A) were grown in S-raffinose –Lys in the presence of Dox for 24 h and then shifted to galactose for the next 24 h to induce Cre expression. Consequently, the cultures were propagated by serial dilutions in YPD in the presence of Dox. DNA extracted from the samples taken at indicated PDs after Cre induction was digested with either PacI and MfeI or XhoI and subjected to Southern blot analyses with either VII-L-specific (see Figure 1A) or TG1–3 probe to visualize VII-L and bulk telomeres, respectively. The pattern of telomeres observed in the Tet-off TLC1 strain during the last four time points is characteristic for type II survivors. (DOCX) [file pgen.1004736.s004.docx]

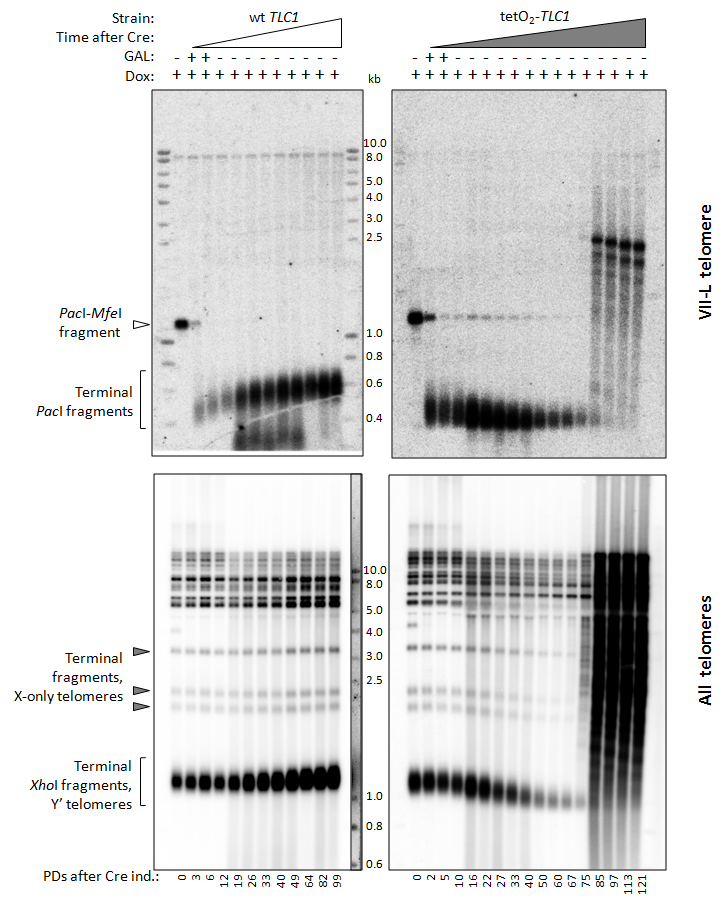


**Figure S4.**  **The effect of *TLC1* repression on VII-L (top panels) and bulk (bottom panels) telomere length.** Wild-type and Tet-off *TLC1* cells with modified TelVII-L (16 Rap1-bs) (see Figure 1A) were grown in S-raffinose –Lys in the presence of Dox for 24 h and then shifted to galactose for the next 24 h to induce Cre expression. Consequently, the cultures were propagated by serial dilutions in YPD in the presence of Dox. DNA extracted from the samples taken at indicated PDs after Cre induction was digested with either *Pac*I and *Mfe*I or *Xho*I and subjected to Southern blot analyses with either VII-L-specific (see Figure 1A) or TG_1-3_ probe to visualize VII-L and bulk telomeres, respectively. The pattern of telomeres observed in the Tet-off *TLC1* strain during the last four time points is characteristic for type II survivors.
