# Supplementary material for: Rad59-Facilitated Acquisition of Y′ Elements by Short Telomeres Delays the Onset of Senescence
Source: PLoS Genet. 2014 Nov 6;10(11):e1004736. doi: 10.1371/journal.pgen.1004736 (PMC4222662; doi:10.1371/journal.pgen.1004736)
Supplement: Figure S5 — Survival of the telomerase-inhibited clones with indicated gene deletions. Tet-off TLC1 strains with indicated gene deletions were grown in the presence of Dox to suppress TLC1 expression. Abrupt shortening of the TelVII-L in “16 Rap1-bs” strain was induced via transient induction of pGAL-Cre by shifting cells to galactose for 24 h. At the end of Cre induction, single cells were micromanipulated on a grid on YPD agar supplemented with Dox. Cell divisions were monitored microscopically and the numbers of cells in microcolonies were counted at 4 and 6 h after plating. Images of the plates taken 3 days after single cells were micromanipulated are shown. Survival graph shows total survival (all micromanipulated cells, blue bars) and survival after arrest (arrested within 8 h after plating, pink bars). (DOCX) [file pgen.1004736.s005.docx]

**Figure S5.** **Survival of the telomerase-inhibited clones with indicated gene deletions.** Tet-off *TLC1* strains with indicated gene deletions were grown in the presence of Dox to suppress *TLC1* expression. Abrupt shortening of the TelVII-L in “16 Rap1-bs” strain was induced via transient induction of pGAL-Cre by shifting cells to galactose for 24 h. At the end of Cre induction, single cells were micromanipulated on a grid on YPD agar supplemented with Dox. Cell divisions were monitored microscopically and the numbers of cells in microcolonies were counted at 4 and 6 h after plating. Images of the plates taken 3 days after single cells were micromanipulated are shown. Survival graph shows total survival (all micromanipulated cells, blue bars) and survival after arrest (arrested within 8 h after plating, pink bars).
