# Supplementary material for: Rad59-Facilitated Acquisition of Y′ Elements by Short Telomeres Delays the Onset of Senescence
Source: PLoS Genet. 2014 Nov 6;10(11):e1004736. doi: 10.1371/journal.pgen.1004736 (PMC4222662; doi:10.1371/journal.pgen.1004736)
Supplement: Figure S6 — Southern blot analysis of the VII-L end in pol32Δ clones. DNA extracted from bulk, B, and clonal, a–f, populations (see caption to Figure 5 for details of the experiment) was digested separately with either PacI or MfeI (restriction sites positions at the VII-L end are shown in the diagram of Figure 2C). Digested DNA was subjected to Southern blot analyses with VII-L-specific probe. The brackets indicate terminal fragments of the telomere VII-L, whereas open arrowheads point to the fragment resulted from VII-L end rearrangement. (DOCX) [file pgen.1004736.s006.docx]

**Figure S6. Southern blot analysis of the VII-L end in *pol32Δ* clones.** DNA extracted from bulk, B, and clonal, a-f, populations (see caption to Figure 5 for details of the experiment) was digested separately with either *Pac*I or *Mfe*I (restriction sites positions at the VII-L end are shown in the diagram of Figure 2C). Digested DNA was subjected to Southern blot analyses with VII-L-specific probe. The brackets indicate terminal fragments of the telomere VII-L, whereas open arrowheads point to the fragment resulted from VII-L end rearrangement.
