# Supplementary material for: Rad59-Facilitated Acquisition of Y′ Elements by Short Telomeres Delays the Onset of Senescence
Source: PLoS Genet. 2014 Nov 6;10(11):e1004736. doi: 10.1371/journal.pgen.1004736 (PMC4222662; doi:10.1371/journal.pgen.1004736)
Supplement: Figure S7 — Southern blot analysis of the TelVII-L state in the randomly chosen rad59Δ and rad51Δ “16Rap1-bs” clones isolated at ∼16 PD after Cre induction. See legend to figure S2 for further details. (DOCX) [file pgen.1004736.s007.docx]

**Figure S7. Southern blot analysis of the TelVII-L state in the randomly chosen *rad59*Δ and *rad51*Δ “16Rap1-bs” clones isolated at ~16 PD after Cre induction.** See legend to figure S2 for further details.
