# Supplementary material for: Rad59-Facilitated Acquisition of Y′ Elements by Short Telomeres Delays the Onset of Senescence
Source: PLoS Genet. 2014 Nov 6;10(11):e1004736. doi: 10.1371/journal.pgen.1004736 (PMC4222662; doi:10.1371/journal.pgen.1004736)
Supplement: Figure S8 — Y′ element translocation on the native telomere VI-R can also be detected by PCR across the junction. (A) Schematic of the primer design for detection of the Y′ translocation onto native VI-R end, which contains only X element. Y′ donor refers to any X and Y′ element containing chromosome end. (B) Semi-quantitative PCR across the VI-R/Y′ junction. DNA was extracted from 2x Cre-loxP strains grown in liquid culture at 0, 10, 50 PD after induction of Cre expression (as in Figures 1B and 3B) and used as a template in PCR reaction with two different pairs of primers designed to amplify the VI-R/Y′ junction. The bracket indicates expected size range of the junction PCR product. TC – template control, a 0.47-kb PCR product amplified at the very terminus of native VI-R end. (DOCX) [file pgen.1004736.s008.docx]

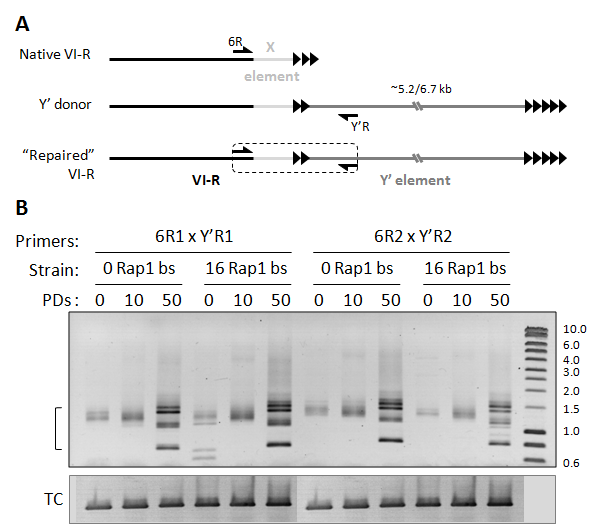


**Figure S8. Y’ element translocation on the native telomere VI-R can also be detected by PCR across the junction.** (A) Schematic of the primer design for detection of the Y’ translocation onto native VI-R end, which contains only X element. Y’ donor refers to any X and Y’ element containing chromosome end. (B) Semi-quantitative PCR across the VI-R/Y’ junction. DNA was extracted from 2x Cre-loxP strains grown in liquid culture at 0, 10, 50 PD after induction of Cre expression (as in Figures 1B and 3B) and used as a template in PCR reaction with two different pairs of primers designed to amplify the VI-R/Y’ junction. The bracket indicates expected size range of the junction PCR product. TC – template control, a 0.47-kb PCR product amplified at the very terminus of native VI-R end.
