# Supplementary material for: Rad59-Facilitated Acquisition of Y′ Elements by Short Telomeres Delays the Onset of Senescence
Source: PLoS Genet. 2014 Nov 6;10(11):e1004736. doi: 10.1371/journal.pgen.1004736 (PMC4222662; doi:10.1371/journal.pgen.1004736)
Supplement: Figure S9 — Rap1 binds the sequences between X and Y′ and between tandem Y′ elements. Rap1 ChIP tiling array data are shown for the terminal 12.5 kb of the selected chromosome ends with the following subtelomere organization: I-L, X element only; II-L, short Y′ and X elements are separated by degenerate TG1–3 repeats; VI-L, short Y′ and X elements are separated by 139 bp of TG1–3 repeats; two short tandem Y′ elements are separated by 64 bp of TG1–3 repeats, and the proximal Y′ and X elements are separated by 163 bp of TG1–3 repeats; XIV-L, long Y′ and X elements are separated by degenerate TG1–3 repeats. The G-rich sequences separating subtelomeric elements were considered degenerate if there were no stretches of TG1–3 longer than 12 bp. Data are plotted for a set range (−0.80–8.40) for all chromosome ends using IGV version 2.3.25. (DOCX) [file pgen.1004736.s009.docx]

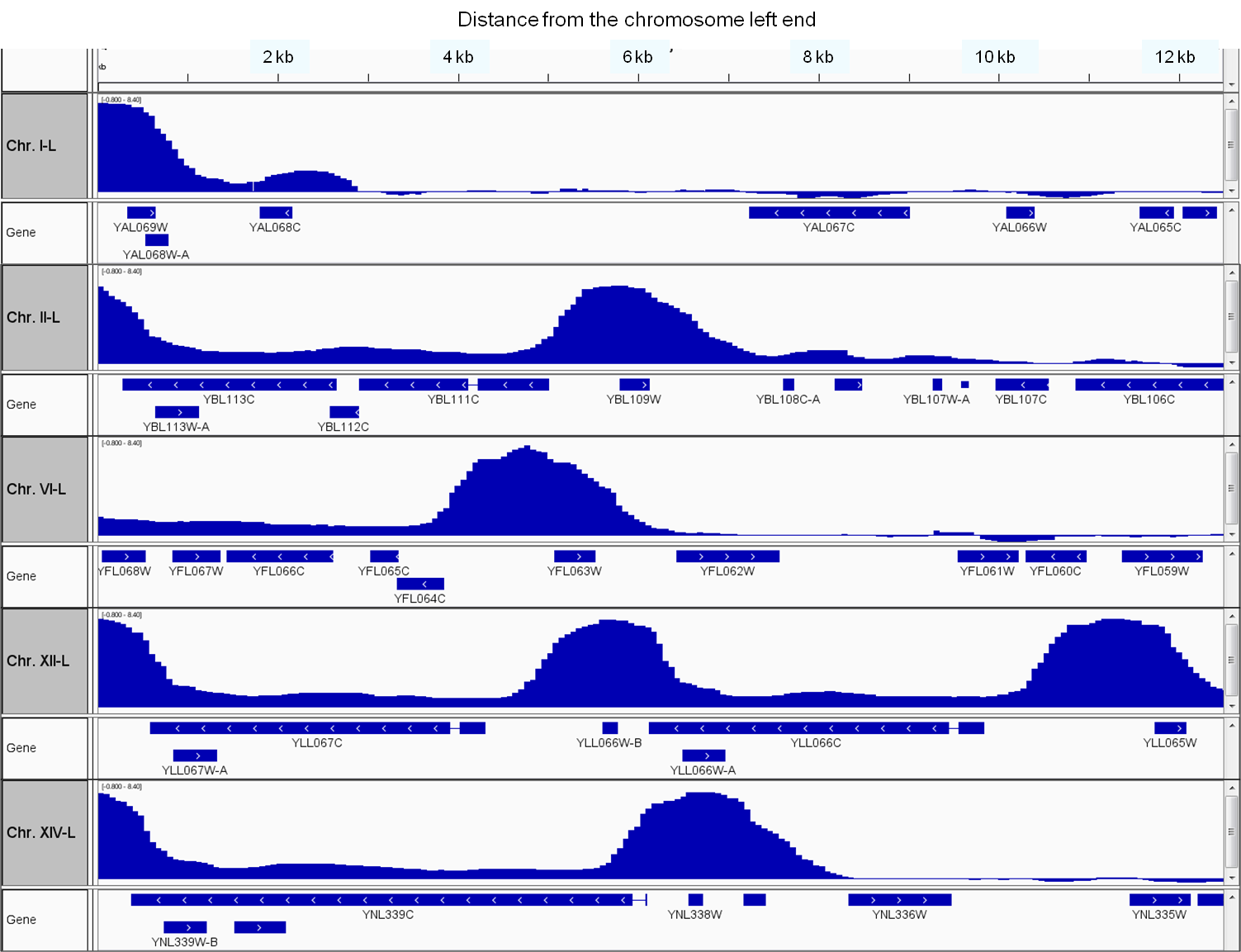


**Figure S9. Rap1 binds the sequences between X and Y’ and between tandem Y’ elements.** Rap1 ChIP tiling array data are shown for the terminal 12.5 kb of the selected chromosome ends with the following subtelomere organization: I-L, X element only; II-L, short Y’ and X elements are separated by degenerate TG_1-3_ repeats; VI-L, short Y’ and X elements are separated by 139 bp of TG_1-3_ repeats; two short tandem Y’ elements are separated by 64 bp of TG_1-3_ repeats, and the proximal Y’ and X elements are separated by 163 bp of TG_1-3_ repeats; XIV-L, long Y’ and X elements are separated by degenerate TG_1-3_ repeats. The G-rich sequences separating subtelomeric elements were considered degenerate if there were no stretches of TG_1-3_ longer than 12 bp. Data are plotted for a set range (-0.80 – 8.40) for all chromosome ends using IGV version 2.3.25.
