# Supplementary material for: Rad59-Facilitated Acquisition of Y′ Elements by Short Telomeres Delays the Onset of Senescence
Source: PLoS Genet. 2014 Nov 6;10(11):e1004736. doi: 10.1371/journal.pgen.1004736 (PMC4222662; doi:10.1371/journal.pgen.1004736)
Supplement: Figure S10 — Rad59 association with TG1–3 repeats during proliferative decline of est2Δ cells. Rad59 binding to terminal (telomeric) and internal (subtelomeric) TG1–3 repeats was determined by ChIP-qPCR during outgrowth of the Rad59-13Myc est2Δ spore clone up to the peak of crisis (maximum decline of the doubling time). Enrichment of the telomere-specific sequences immunoprecipitated with Rad59 was determined relative to non-specific locus as follows: fold enrichment = (TeloChIP/TeloInput)/(GAL2 ChIP/GAL2 Input). (A) Rad59 ChIP at individual X-only telomeres. (B) Rad59 ChIP at an average X-only telomere (mean ±SE, n = 4) and at the internal sequences between X and Y′ elements. (DOCX) [file pgen.1004736.s010.docx]

**Figure S10. Rad59 association with TG_1-3_ repeats during proliferative decline of *est2Δ* cells.** Rad59 binding to terminal (telomeric) and internal (subtelomeric) TG_1-3_ repeats was determined by ChIP-qPCR during outgrowth of the *Rad59-13Myc est2Δ* spore clone up to the peak of crisis (maximum decline of the doubling time). Enrichment of the telomere-specific sequences immunoprecipitated with Rad59 was determined relative to non-specific locus as follows: fold enrichment = (Telo_ChIP_/Telo_Input_)/ (*GAL2*_ChIP_/*GAL2*_Input_). (A) Rad59 ChIP at individual X-only telomeres. (B) Rad59 ChIP at an average X-only telomere (mean ± SE, n=4) and at the internal sequences between X and Y’ elements.
