# Supplementary material for: Rad59-Facilitated Acquisition of Y′ Elements by Short Telomeres Delays the Onset of Senescence
Source: PLoS Genet. 2014 Nov 6;10(11):e1004736. doi: 10.1371/journal.pgen.1004736 (PMC4222662; doi:10.1371/journal.pgen.1004736)
Supplement: Figure S11 — SAE2 deletion substantially reduces the efficiency of type II survivor formation. (A) est2Δ sae2Δ mutants generate predominantly type I survivors, as opposed to est2Δ, which generate mostly type II survivors in liquid culture. Only the clones that survived via type I pathway were included in the comparison with triple est2Δ sae2Δ rad59Δ mutants (see Figure 8A and 8B), which generate exclusively type I survivors. DNA was extracted at the indicated days during senescence time course, digested with XhoI, subjected to Southern blot, and probed for TG1–3 repeats. The graphs above the Southern blots show the growth profiles during senescence in liquid cultures for indicated genotypes. (B) Histogram showing the fractions of different survivor types generated by the indicated genotypes. (DOCX) [file pgen.1004736.s011.docx]

**Figure S11. *SAE2* deletion substantially reduces the efficiency of type II survivor formation.** (A) *est2Δ sae2Δ* mutants generate predominantly type I survivors, as opposed to *est2Δ*, which generate mostly type II survivors in liquid culture (data not shown). Only the clones that survived via type I pathway were included in the comparison with triple *est2Δ sae2Δ rad59Δ* mutants (see Figure 8A and 8B), which generate exclusively type I survivors. DNA was extracted at the indicated days during senescence time course, digested with *Xho*I, subjected to Southern blot, and probed for TG_1-3_ repeats. The graphs above the Southern blots show the growth profiles during senescence in liquid cultures for indicated genotypes. (B) Histogram showing the fractions of different survivor types generated by the indicated genotypes.
