# Supplementary material for: Rad59-Facilitated Acquisition of Y′ Elements by Short Telomeres Delays the Onset of Senescence
Source: PLoS Genet. 2014 Nov 6;10(11):e1004736. doi: 10.1371/journal.pgen.1004736 (PMC4222662; doi:10.1371/journal.pgen.1004736)
Supplement: Table S1 — Primers used in this study. (DOCX) [file pgen.1004736.s012.docx]

Table S1. Primers used in this study

| **Name** | **Sequence** | **Annealing site location** |
| --- | --- | --- |
| *Modified VII-L end-specific probe:* | | |
| A7Lprb1A | 5’- TTAATTAAGAATACGCTGGTTTGC | Modified VII-L end (non-yeast origin) |
| A7Lprb1B | 5’- ACGCGTCCCGGGACAAAATG | Modified VII-L end (non-yeast origin) |
| Subtelomere XV-L probe | | |
| XVLprobeF | 5’-GCCGTACAACGAAGCACAAAG | 2.14 kb from TG_1-3_ repeats |
| XVLprobeR | 5’-ATTGGCTGCTGAAATGCTGCC | 2.55 kb from TG_1-3_ repeats |
| *PCR across translocation junction:* | | |
| 7L1 | 5’-TGGCCTTAGCATTGTCGTGAGC | VII-L end, *ADH4* |
| 6R1 | 5’-TTCGAACGTGATCCTAACGAGTG | VI-R end, adjacent to X element |
| Y’ R1 | 5’-CTATCTGCTTAGTCGAGGAGAAC | Any Y’ element, CEN-proximal end |
| 7L2 | 5’-CAATGGCCTTAGCATTGTCG | VII-L end, *ADH4* |
| 6R2 | 5’-TTAACAAGCGGCTGGACTAC | VI-R end, adjacent to X element |
| Y’ R2 | 5’-TTTCAATTTCCATGGTGCACAG | Any Y’ element, CEN-proximal end |
| *ChIP-qPCR:* | | |
| ChrIL-F | 5’-GGCCAACCTGTCTCTCAACTT | I-L, 38 bp from TG_1-3_ repeats |
| ChrIL-R | 5’-TGGTAGGGTAAGCACGTGTG | I-L, 289 bp from TG_1-3_ repeats |
| FSB11 | 5’-CGTATGCTAAAGTATATATTACTTCACTCCATT | VI-R, 30 bp from TG_1-3_ repeats |
| RSB11 | 5’-TCCGAACTCAGTTACTATTGATGGAA | VI-R, 83 bp from TG_1-3_ repeats |
| ChrXIL-F | 5’-GGTGAGTATGGCATGTGATGTGTG | XI-L, 24 bp from TG_1-3_ repeats |
| ChrXIL-R | 5’-CCTGATCAACCTGTCTCCAAACCTA | XI-L, 325 bp from TG_1-3_ repeats |
| FSB25 | 5’-AACCCTGTCCAACCTGTCTCC | XV-L, 12 bp from TG_1-3_ repeats |
| RSB25 | 5’-ATCGTGGTTCGCTGTGGTAT | XV-L, 87 bp from TG_1-3_ repeats |
| Y’J-F1 | 5’-GACATATAGCATCTAGGAAG | Any Y’, ~50 bp from CEN-proximal end |
| Y’J-R1 | 5’-AAATTCCTTGACGCTAGATC | Any Y’, ~150 bp from CEN-proximal end |
