# Supplementary material for: Rad59-Facilitated Acquisition of Y′ Elements by Short Telomeres Delays the Onset of Senescence
Source: PLoS Genet. 2014 Nov 6;10(11):e1004736. doi: 10.1371/journal.pgen.1004736 (PMC4222662; doi:10.1371/journal.pgen.1004736)
Supplement: Table S2 — Yeast strains used in this study. (DOCX) [file pgen.1004736.s013.docx]

Table S2. Yeast strains used in this study

| **Strain** | **Genotype** | **Source** |
| --- | --- | --- |
| YAB892 | *MAT a, ade2-1 his3-11,15 LEU2-Pgal-Cre lys2::hphMX3 telVIIL-16Rap1bs-LYS2 trp1-1 ura3-1 can1-100 RAD5* | 1. Bianchi |
| YAB893 | *MAT a, ade2-1 his3-11,15 LEU2-Pgal-Cre lys2::hphMX3 telVIIL-0Rap1bs-LYS2 trp1-1 ura3-1 can1-100 RAD5* | 1. Bianchi |
| YAB892 DC | YAB892, *est2::KanMX* +pDS381(*EST2*/*ADE2*) | This study |
| YAB893 DC | YAB893, *est2::KanMX* +pDS381(*EST2*/*ADE2*) | This study |
| YAB892 Tet-off *TLC1* | YAB892, *tetO_2_-TLC1::KanMX* | This study |
| YAB893 Tet-off *TLC1* | YAB893, *tetO_2_-TLC1::KanMX* | This study |
| YAB892 Tet-off *TLC1 rad52Δ* | YAB892 Tet-off *TLC1*, *rad52::TRP1* | This study |
| YAB893 Tet-off *TLC1 rad52Δ* | YAB893 Tet-off *TLC1*, *rad52::TRP1* | This study |
| YAB892 Tet-off *TLC1 rad51Δ* | YAB892 Tet-off *TLC1*, *rad52::TRP1* | This study |
| YAB893 Tet-off *TLC1 rad51Δ* | YAB893 Tet-off *TLC1*, *rad51::TRP1* | This study |
| YAB892 Tet-off *TLC1 rad59Δ* | YAB892 Tet-off *TLC1*, *rad59::TRP1* | This study |
| YAB893 Tet-off *TLC1 rad59Δ* | YAB893 Tet-off *TLC1*, *rad59::TRP1* | This study |
| YAB892 Tet-off *TLC1 pol32Δ* | YAB892 Tet-off *TLC1*, *pol32::TRP1* | This study |
| YAB893 Tet-off *TLC1 pol32Δ* | YAB893 Tet-off *TLC1*, *pol32::TRP1* | This study |
| MNY1292 | *est2::LEU2 rad59::KAN sae2::TRP* | M.-N. Simon |
| JHY221 | *est2::LEU2 sgs1::KAN sae2::TRP* | M.-N. Simon |
| MNY1335 | *est2::LEU2 mec1::HIS sml::KAN TelVIR-8xlexA BS* | M.-N. Simon |
